# Supplementary material for: RMPJ: An ImageJ plugin for morphological information processing in biomedical images
Source: PLoS Comput Biol. 2025 Apr 16;21(4):e1012992. doi: 10.1371/journal.pcbi.1012992 (PMC12037072; doi:10.1371/journal.pcbi.1012992)
Supplement: S1 Text — (PDF) [file pcbi.1012992.s001.pdf]

RMPJ: An ImageJ plugin for morphological information processing in biomedical images

Theoretical background of mathematical morphology and rotational morphological processing (Supporting Information 1)

Yoshitaka Kimori

1. Theoretical background

1.1. Mathematical morphology

In mathematical morphology, a grayscale image is represented as a function $f(x,y)$ of two-dimensional spatial coordinates x and y. The amplitude f is the intensity value at point (x,y). By projecting the spatial coordinates onto the $x-y$ plane and mapping the remaining dimension to the intensity values, the function $f(x,y)$ can be represented as a point $(x,y,f(x,y))$ in a three-dimensional space. The function $f(x,y)$ can be considered a binary set in a three-dimensional space.

The basic operations in mathematical morphology include dilation and erosion. The dilation (δ) of f by a binary structuring element (SE) B is defined as Eq (S1), and the erosion (ε) of f by a binary SE B is defined as Eq (S2).

$$[\delta_B(f)](x,y) = \max_{(s,t)\epsilon B} f(x-s, y-t), \qquad \text{(S1)}$$

$$[\varepsilon_B(f)](x,y) = \min_{(s,t)\epsilon B} f(x+s, y+t), \qquad \text{(S2)}$$

where (s,t) are the coordinates of the SE B.

Dilation replaces the intensity value of the coordinates of interest (x,y) with the maximum intensity value within the neighborhood of the grayscale image centered on that coordinate. The SE defines the size and shape of this neighborhood. However, in erosion, the operation of selecting the maximum value considering dilation is replaced by the selection of the minimum value.

By combining the operations of dilation and erosion, new operations of opening and closing are formed. The opening and closing operations are used to smooth the image. If there exist convex regions (regions with higher intensity values than the surrounding regions) in an image with a width

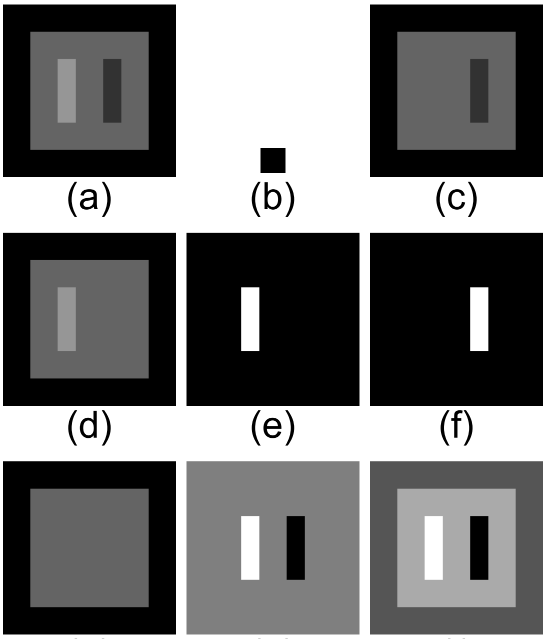

Fig. S1. Examples of morphological processing. (a) Original image. (b) Square-shaped structural element (SE). (c) to (i) are the results of applying different morphological processing methods to the original image (a). The SE (b) was used in all processing operations: (c) opening, γ; (d) closing, ϕ; (e) white top-hat, WTH; (f) black top-hat, BTH; (g) morphological smoothing, MS; (h) morphological contrast enhancement type1, MCE_1; and (i) morphological contrast enhancement type2, MCE_2.

smaller than the size of the SE, these regions are removed using the opening operation. Consequently, the image is smoothed by removing fine protrusions. However, if there are concave regions (regions with lower intensity values than the surrounding regions) in an image with a width smaller than the size of the SE, the closing operation fills them, resulting in a smooth image. For an image f, opening γ with SE B is defined by Eq (S3) and its dual operation, closing ϕ, by Eq (S4). In opening, the dilation operation is performed after the erosion operation, whereas in closing, the erosion operation is performed after the dilation operation.

$$\gamma_B(f) = \delta_B[\varepsilon_B(f)], \tag{S3}$$

$$\phi_B(f) = \varepsilon_B[\delta_B(f)]. \tag{S4}$$

Figs. S1(c) and S1(d) depict the outcomes of the opening and closing operations applied to the original image (Fig. S1(a)), respectively. The square-shaped SE (Fig. S1(b)) was used for each operation.

Morphological top-hats are operators that extract structures that are removed by opening and closing. The process of extracting convex regions (regions with high-intensity values compared to their surroundings) from an image is referred to as the white top-hat (WTH) operation. The WTH of an image f is the arithmetic difference between f and its opening γ:

$$WTH(f) = f - \gamma(f). \tag{S5}$$

In contrast, the operation to extract concave regions (regions with low-intensity values compared to their surroundings) is referred to as the black top-hat (BTH). The BTH of an image f is the arithmetic difference between the closing of f and f:

$$BTH(f) = \phi(f) - f. \tag{S6}$$

The results of applying WTH and BTH to the original image f are illustrated in Figs. S1(e) and S1(f), respectively.

A morphological smoothing filter has been proposed that uses a linear combination of opening–closing and closing–opening operations [1], which is denoted as MS and is defined as follows:

$$MS(f) = \frac{1}{2}[\gamma(\phi(f))] + \frac{1}{2}[\phi(\gamma(f))]. \tag{S7}$$

Fig. S1(g). depicts the results of smoothing the original image. By averaging the pixel values of the output of the opening operation applied to the closed image of f and the closing operation applied to the open image of f, a more effective smoothing result with less biased intensity values can be obtained as compared to when using the opening and closing operations separately.

The aforementioned morphological operators can be used to create contrast enhancement methods. Two types of morphological contrast enhancement filters, morphological contrast enhancement type 1 (MCE_1) [2] and morphological contrast enhancement type 2 (MCE_2) [3], are defined as follows:

$$MCE_1(f) = f - MS(f). \tag{S8}$$

$$MCE_2(f) = f + WTH(f) - BTH(f). \tag{S9}$$

Eq (S8) demonstrates the difference between the original image f and the image after applying morphological smoothing MS. The structures, which were removed by smoothing, were extracted. Fig. S1(h) displays the outcome of applying MCE_1 to the original image f. In addition, Eq (S9)

enhances the structures extracted by the white and black top-hat operations. The application of MCE_2 to the original image f is illustrated in Fig. S1(i). Owing to differencing, certain pixels exhibit negative intensity values. Therefore, the dynamic ranges of the processed images must be modified.

1.2. Rotational morphological processing (RMP)

Traditional mathematical morphology has certain drawbacks in terms of preserving the shape of the object to be analyzed in the original image. Specifically, when morphological operations are applied, the shape of the object may be deformed owing to the shape of the SE.

Fig. S2(a) depicts an image of two rectangular objects placed on a background consisting of round structures. This represents the original image. The rectangular object on the right side of the image corresponds to the rectangular object on the left side, rotated 45° clockwise, with its center of gravity as the origin. These rectangles are considered the objects of analysis, and the round structures are considered noise. An opening operation is applied to the original image to remove this noise. The structures of the two rectangles must be preserved even when the opening operation is applied.

The basic effect of the opening operation is to preserve the regions in the image, where the SE fits, and to remove the regions, where it does not fit.

Fig. S2(b) depicts the resulting image after applying an opening operation with a square SE to the original image. All the round structures are eliminated; however, the rectangular structure on the right side is distorted. Fig. S2(c) depicts some of the positions of the SE during the opening operation (in practice, the SE moves from the top left to the bottom right of the image, one pixel at a time, as a raster scan, and a morphological operation is performed each time it moves). The set of positions where the SE fits the rectangular object is highlighted in cyan, whereas the set of positions, where it does not fit, is highlighted in magenta. The SE could fit into any region of the left rectangular object; thus, the entire shape of the object is preserved. Because the round structures are smaller than the SE, no regions exist, where the SE could fit; therefore, all round structures are eliminated. Additionally, the four corners of the rectangular object on the right side did not fit the SE; therefore, these regions were removed. Considering this example, the structure of the object to be analyzed may be deformed by morphological operations, depending on its orientation.

To address this problem, a method called rotational morphological processing (RMP) was introduced. The basic concept is as follows: first, the image containing the target object is rotated in the direction that fits the SE. Next, the morphological operation is applied to each of the rotated images. Finally, the images after the operation are combined and output as a single image.

In this example, if the image is rotated 45° and the opening operation is applied to it, the overall structure of the rectangular object on the right is preserved (Fig. S2(d)). This time, the four corners of the rectangular object on the left side are missing. However, by combining the two processed images, the original shape of the two rectangular objects is restored. RMP applies a process that rotates the

original image at different angles to enhance the probability of fitting the SE to the structure of the object to be analyzed with various orientations.

Fig. S2(e) illustrates the process of opening by RMP to the original image (denoted as f in Fig. S2(a)). The original image f is first rotated clockwise by θ degrees, which corresponds to the forward direction. Let θ = 0° and 45°; f_1 denotes the image with f rotated by 0° and f_2 denotes the image with f rotated by 45°. The opening operation is then applied to the images. The resulting

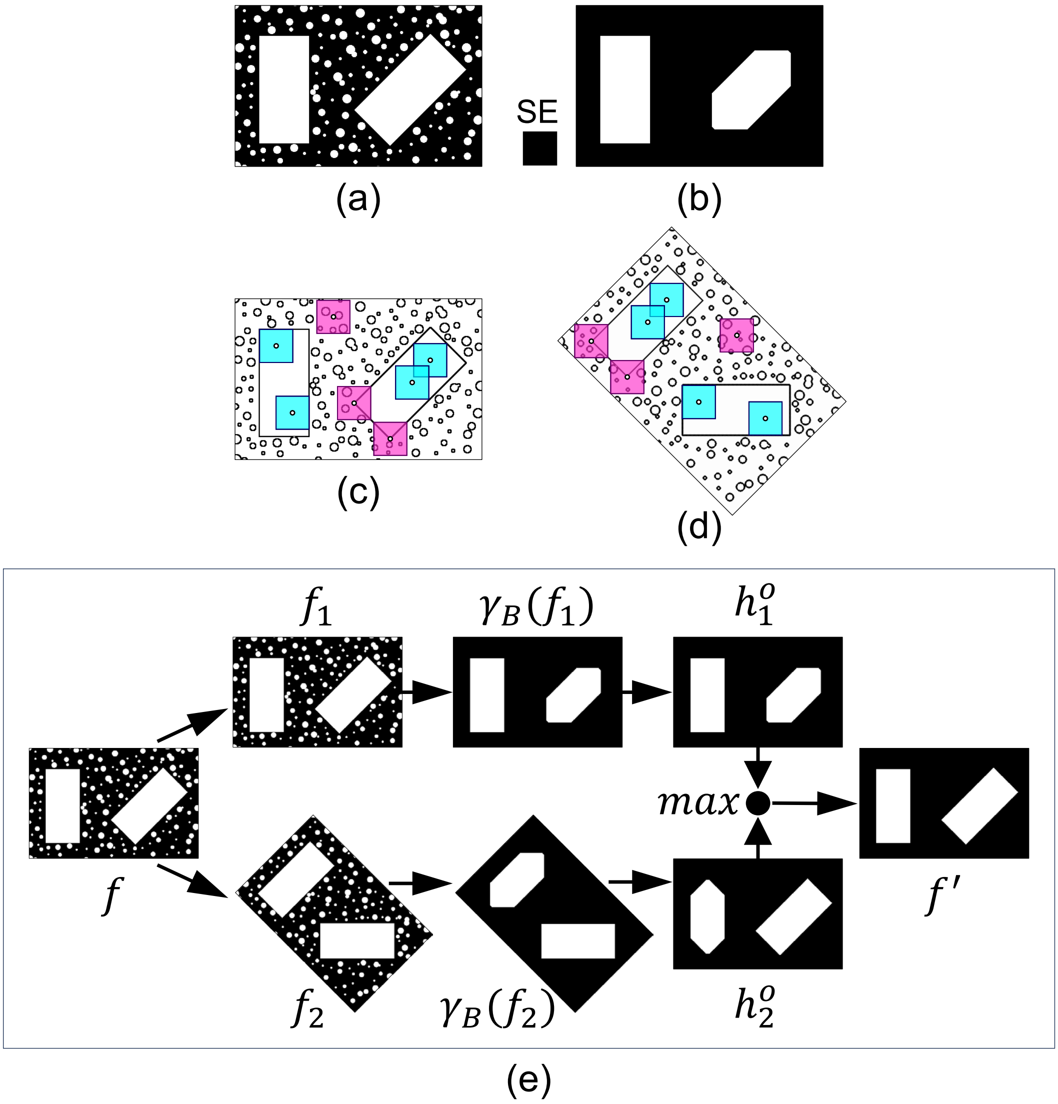

Fig. S2. Concept of RMP. (a) Original image. (b) Resulting image of the opening operation. (c) and (d) Display of some positions of SE during the opening operation. The SE is represented by cyan if it is located in a position that fits into the region of the rectangular object and by magenta if it is located in a position that does not fit. (e) Process of opening by RMP.

images are denoted as $\gamma_B(f_1)$ and $\gamma_B(f_2)$. The opened images are rotated counterclockwise by θ degrees. Specifically, $\gamma_B(f_1)$ is rotated 0° and $\gamma_B(f_2)$ is rotated by -45°. The rotated images are denoted as h_1^o and h_2^o. Finally, the rotated images are combined and output as a single image. In the case of opening by RMP, the maximum operation is applied to calculate the maximum value from the intensity values of pixels at the same coordinates in all rotated images. The rotated images h_1^o and h_2^o are combined and output through opening by RMP f'.

Applying the opening operation to f_1 deforms the structure of the right rectangle (h_1^o), whereas applying it to f_2 deforms the structure of the left rectangle (h_2^o). However, the missing corners of each rectangle are restored by combining these images. The resulting image demonstrates that noise can be eliminated while preserving the objects to be analyzed.

As generally, objects to be analyzed are oriented in various directions, the original image must be rotated to various angles for practical use. Subsequently, a morphology operation must be applied to each rotated image.

Let *N* denote the number of image rotations. Then, let α be the value obtained by dividing 180, the maximum value of the range of rotation angles, by *N*, i.e., $\alpha = \frac{180}{N}$. To perform the *i*-th morphological operation, the angle at which the original image *f* is rotated is defined as follows:

$$\theta_i = \alpha(i-1), \tag{S10}$$

where $i = 1, 2, \cdots, N$ and $\theta_i \in [0,180)°$. The result of the morphology operation on an image rotated by 180° is identical to the 0° case; therefore, the 180° angle is not included.

Previous experiments [4] have demonstrated that the optimal value of *N* is *N* = 8 ($\theta_1 = 0°$, $\theta_2 = 22.5°$, $\theta_3 = 45°$, …, $\theta_8 = 157.5°$) when using a square- or disk-shaped SE and *N* = 36 ($\theta_1 = 0°$, $\theta_2 = 5°$, $\theta_3 = 10°$, …, $\theta_{36} = 175°$) when using a line-shaped SE for morphological operations.

The opening by RMP with SE B for original image f is defined as follows:

$$[\gamma_B^R(f)](x,y) = \max_{i \in (1,2,\ldots,N)} h_i^o(x,y). \tag{S11}$$

This process is shown in Fig. S3.

The closing by RMP, with SE B for the original image f, is defined as follows:

$$[\phi_B^R(f)](x,y) = \min_{i \in (1,2,\ldots,N)} h_i^c(x,y), \tag{S12}$$

where h_i^c is the image that is rotated counterclockwise by θ_i from the closed image (which is the

result of applying the closing operation to a rotated image that is rotated clockwise by θ_i from the original image). In closing by RMP, the minimum operation is applied to combine the rotated images. Fig. S4 provides a detailed description of this process.

The white top-hat of the RMP (WTH^R) and the black top-hat of the RMP (BTH^R) are defined using these operations:

$$WTH^R(f) = f - \gamma^R(f), \tag{S13}$$

$$BTH^R(f) = \phi^R(f) - f. \tag{S14}$$

Details of the application of these RMP-based operations, including examples, are described in references [4] and [5].

The RMP version of the smoothing filter MS (Eq (S7)), MS^R, is defined in Eq (S15) [2]. In addition, MCE_1^R, which is the RMP version of image enhancement filter MCE_1 (Eq (S8)), is defined in Eq (S16) [2], and MCE_2^R, which is the RMP version of image enhancement filter MCE_2 (Eq (S9)), is defined in Eq (S17) [6].

$$MS^R(f) = \frac{1}{2}[\gamma^R(\phi^R(f))] + \frac{1}{2}[\phi^R(\gamma^R(f))], \tag{S15}$$

$$MCE_1^R(f) = f - SM^R(f), \tag{S16}$$

$$MCE_2^R(f) = f + WTH^R(f) - BTH^R(f). \tag{S17}$$

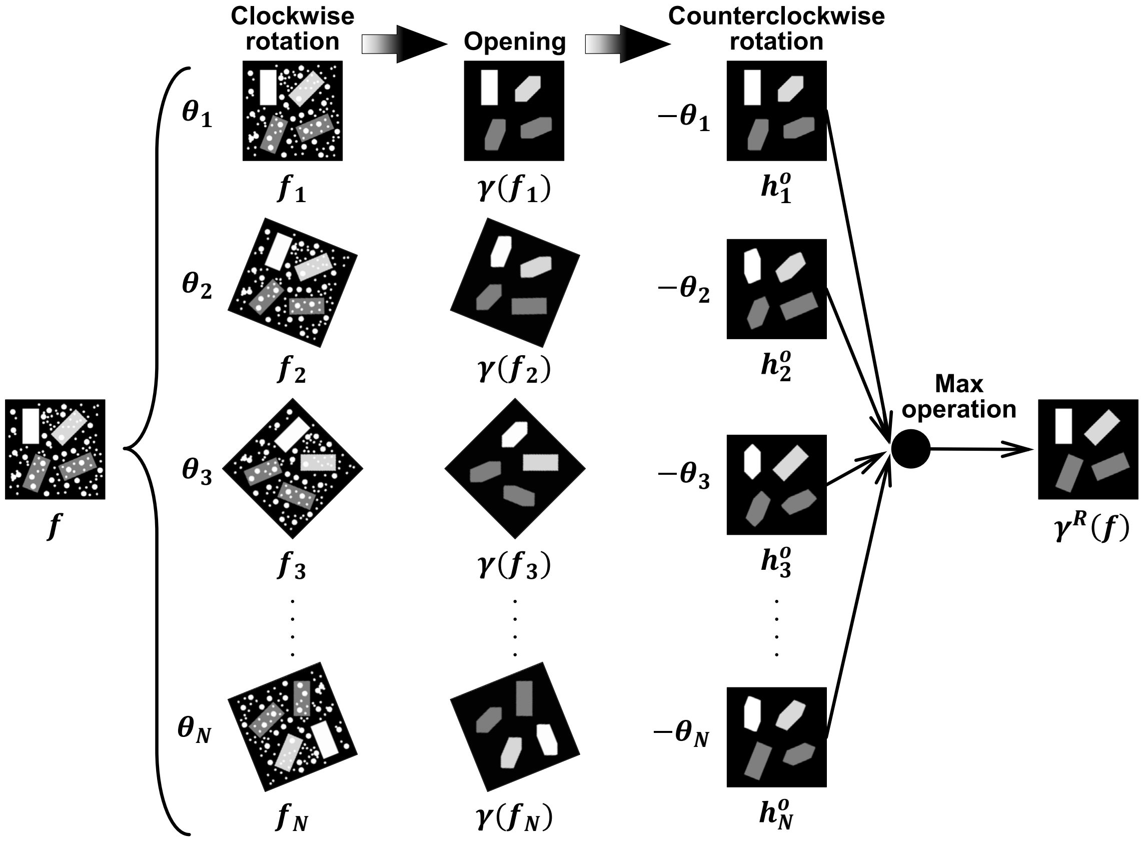

Fig. S3. Process flow for opening by RMP. The original image f consists of four square objects and noise. First, the original image is rotated clockwise (the rotation angles are θ_1, θ_2, … , θ_N). Subsequently, the opening operation is applied to each of these rotated images. Then, these opening images are rotated counterclockwise (the rotation angles are $-\theta_1$, $-\theta_2$, … , $-\theta_N$). Finally, these images are combined into a single image using a maximum operation. Opening by RMP enables noise reduction that preserves the shape of the four square objects.

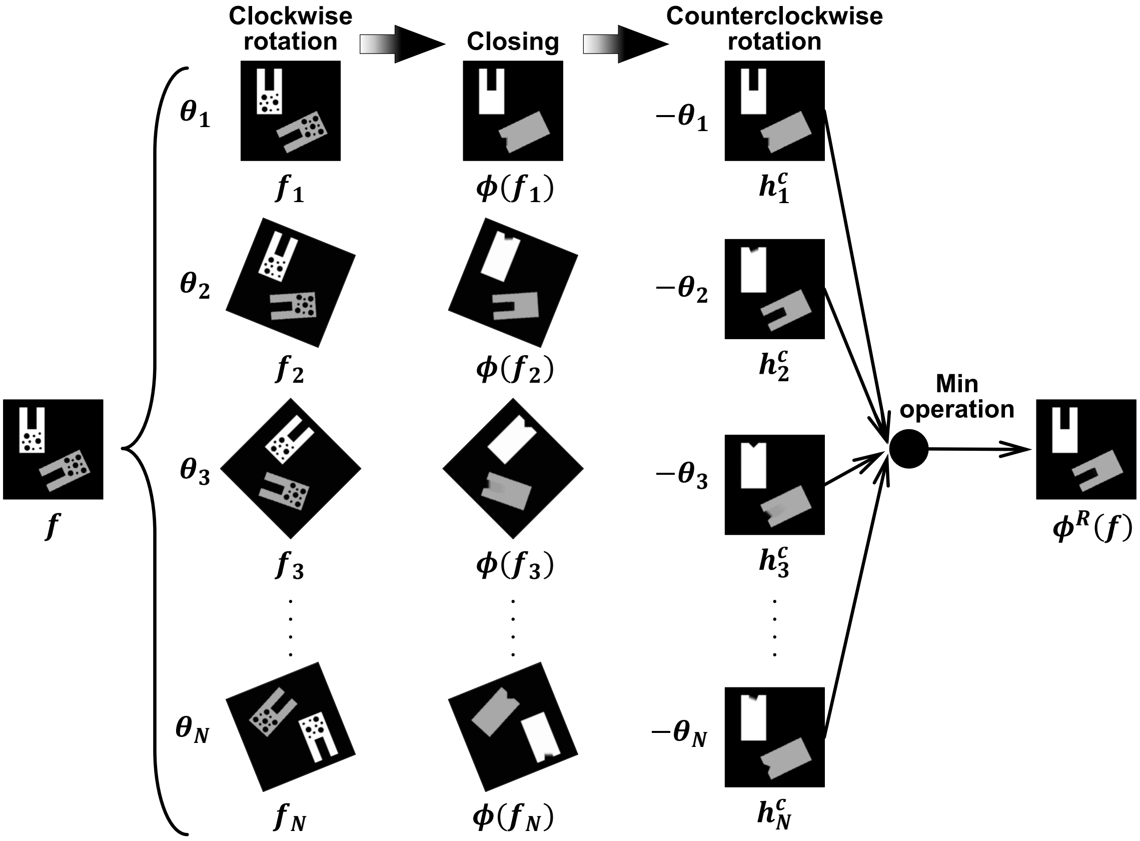

Fig. S4. Process flow for closing by RMP. In the original image f, two target objects and several holes in their interior regions exist. Consider filling these holes using the closing operation. First, the original image is rotated clockwise (the rotation angles are θ_1, θ_2, … , θ_N). Next, the closing operation is applied to each of these rotated images. Then, these closing images are rotated counterclockwise (the rotation angles are $-\theta_1$, $-\theta_2$, … , $-\theta_N$). Finally, these images are combined into a single image using a minimum operation. Using closing by RMP, the holes in the interior region of the target object can be filled without any deformation to its shape.

2. Illustrative examples

This section demonstrates the efficacy of the RMP through morphological image processing examples.

2.1. Examples of morphology operations with line-shaped SE: separation and extraction of aggregated particles

Biological image processing necessitates the separation of aggregated particles and their extraction as individual particles. WTH^R with a line-shaped SE is demonstrated to satisfy this requirement (see Figs. 3(a) and 3(c), in main text).

Fig. S5(a) (top) depicts the original image (256 × 256 pixels) of aggregated particles on a background with an intensity gradient, whereas Fig. S5(a) (middle) shows the binarized original image

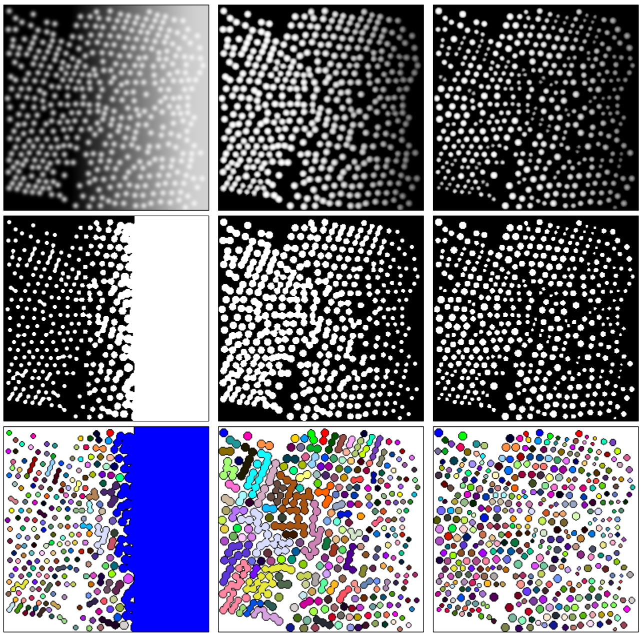

(a) (b) (c)

Fig. S5. Separation and extraction of aggregated particles by WTH^R. (a) Original image (top), binarized image (middle), and image with regions of extracted particles highlighted in different colors (bottom). (b) Result of applying WTH^R with a disk-shaped SE to the original image (top) and its binarized image (middle). Image with regions of extracted particles highlighted in different colors (bottom). (c) Result of applying WTH^R with a line-shaped SE to the original image (top) and its binarized image (middle). Image with regions of extracted particles highlighted in different colors (bottom).

obtained using Otsu's method [7]. The extracted particles are highlighted in different colors (Fig. S5(a) (bottom)). Particles with high-intensity contrast to the background that are relatively distant from neighboring particles are extracted. However, the aggregated particles and particles with low contrast to the background are not separated or extracted. Fig. S5(b) (top) shows the results of applying WTH^R with a disk-shaped SE (size: 11 pixels) to the original image ($N = 8$). The background intensity gradient is removed and the particles are emphasized. Fig. S5(b) (middle) shows the binarized results of Fig. S5(b) (top), and Fig. S5(b) (bottom) shows the extracted particles in different colors. The results indicate that the aggregated particles are extracted as a single region without being separated. The diameter of the disk-shaped SE is larger than the width of the valley between the peaks of adjacent particles; therefore, this SE does not fit into its region during the opening operation. Consequently, the top-hat transformation leaves the valley region as a residual. This aspect is true even for WTH^R. Therefore, the particles in close proximity cannot be separated.

Fig. S5(c) (top) depicts the outcome of applying WTH^R with a line-shaped SE (size: 11 pixels) to the original image ($N = 36$), whereas Fig. S5(c) (middle) depicts the binarized image. Fig. S5(c) (bottom) shows the extracted particles highlighted in different colors. The results demonstrate that all

particles are separated and extracted. The width of the line-shaped SE is one pixel; therefore, it can enter regions where the particles are in contact with each other. Consequently, particles in close proximity can be separated.

2.2. Application to stacked image data

This section presents an example of stacked data processing (BBBC034v1, Thirstrup et al. 2018) obtained from the Broad Bioimage Benchmark Collection [8]. Filament structures were extracted from channel 2 (AICS-12_134_C=1) image data and reconstructed in 3D.

First, a Gaussian blur is applied to the original images (Fig. S6(a)) after contrast normalization. These images are then converted to 8-bit grayscale images. Subsequently, WTH^R ($N = 8$) is applied to these grayscale images using a disk-shaped SE (size: 11 pixels) to enhance the filament structures (Fig. S6(b)). The resulting images are binarized using Otsu's automatic thresholding method (Fig. S6(c)). Fig. S6(d) shows the 3D reconstruction results obtained from the stacked binarized images. UCSF Chimera [9] was used for the 3D reconstruction and visualization.

The results demonstrate that filament structures of different intensities and orientations are effectively enhanced by RMPJ and that the binarization process can successfully extract these filaments. Such image processing using RMPJ enables the extraction of complex structures that comprise a biological sample and analysis of the characteristics of its 3D structures.

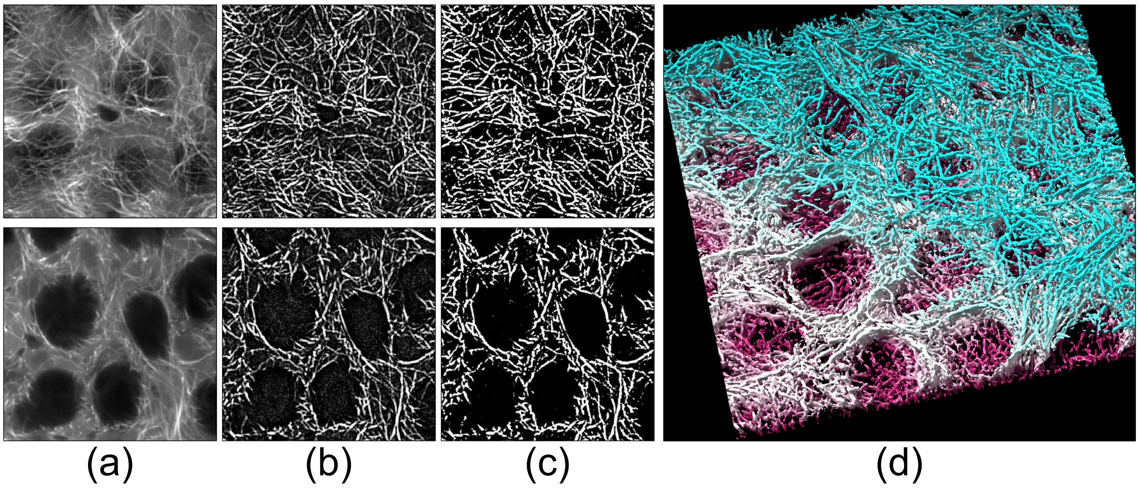

Fig. S6. Extraction of filament structure from the stack image. (a) Images obtained from the BBBC034v1 dataset (AICS_12_134_C=1.tif). The 10th (top) and 15th (bottom) images in the stack are shown. These are partial images of the entire image. (b) Filament structure enhancement results from the application of WTH^R. (c) Binarized images of those shown in (b). (d) 3D reconstruction result.

2.3. Example of extraction of filamentous structures: extraction of crack region in road pavement

One of the main purposes of applying image processing using mathematical morphology is to extract thin structures such as filamentous structures. In Fig. 2 of the main text, examples of structures such as mammary glands in the breast region are emphasized using image enhancement filters such as the top-hat filter.

This section shows an example of extracting filamentous structures based on smoothing filters. For this experiment, the dataset CRACK500 [10] was used, which is a collection of images of cracks in road pavements. The top of Fig. S7(a) is an image created by converting the RGB color image in this data set, file name "DeepCrack_11215-14", to grayscale (this RGB color image was converted to HSV color space, and then the brightness channel was extracted. This was used as the grayscale image to be processed) and then inverting the image intensity. The image size has been resized to 448×448 pixels. This image is used as the original image. From this original image, an attempt is made to extract the crack region, which is a filamentous structure. The ground truth for the crack region (data set file name: "DeepCrack_11215-14_GT") is shown at the bottom of Fig. S7(a).

The results of applying WTH^R (N = 36) using a line-shaped SE with a length of 17 pixels to the original image are shown at the top of Fig. S7(b). From this result image, we can observe that the granular structures of the background are emphasized more than the crack region. The binary image created using Otsu's method is shown at the bottom of Fig. S7(b). The emphasized granular structures that exist in the background are extracted. By contrast, most structures in the crack region are not extracted. This is because the line-shaped SE fits most of the crack structures.

In such cases, the use of smoothing filters may be useful. The strategy is to remove the background structures by smoothing, enabling the background to be represented with homogeneous intensity. Then, binarization is used to extract filamentous structures. In this case, maintaining the filamentous structure as much as possible without destroying is necessary, even when applying smoothing processing. For this purpose, smoothing operations based on RMP with line-shaped SE are effective. Using line-shaped SE can preserve the filamentous structure because line-shaped SE fits most of the filamentous structure.

The top of Fig. S7(c) shows the result of applying γ^R (N = 36) using a line-shaped SE with a length of 17 pixels to the original image. We can observe that the background granular structures have been removed. The structure of the crack region is well preserved. The bottom of Fig. S7(c) shows the result of binarizing that opening image.

Furthermore, at the top of Fig. S7(d), the result of applying MS^R (N = 36) with a line-shaped SE of 17 pixels in length to the original image is shown. Compared to the image after applying γ^R, the background of this result image is expressed with a more uniform intensity. Additionally, the crack structure is well preserved. The bottom of Fig. S7(d) is the result of binarizing the image produced by

MS^R. The binary images in Figs. S7(c) and S7(d) are extremely similar to the ground truth. This shows that the γ^R and MS^R operations can be used effectively to extract crack regions. In the case of the results using γ^R, the parts with low intensity in the crack region remain as "holes" in the binary image. However, in the case of the results using MS^R, these holes are filled in.

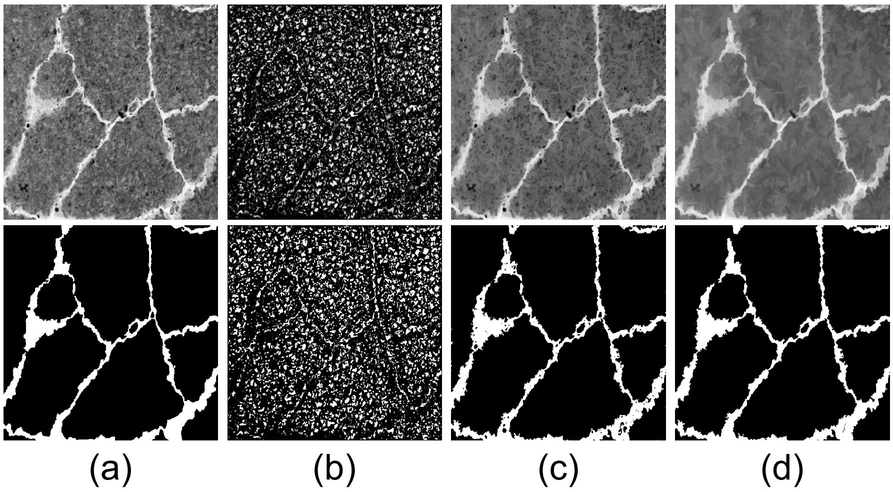

Fig. S7. Extraction of crack regions in road pavement. (a) Original image (top), ground truth (bottom). (b) Resulting image of WTH^R using line-shaped SE on the original image (top). Binarized image of the resulting image of WTH^R (bottom). (c) Resulting image of γ^R using line-shaped SE on the original image (top). Binarized image of the resulting image of γ^R (bottom). (d) Resulting image of MS^R using line-shaped SE on the original image (top). Binarized image of the resulting image of MS^R (bottom).

2.4. Selection of the shape of SE and determination of the number of image rotations (*N*)

The shape of the SE used in operations based on RMP is selected according to the structure of the object being analyzed. The most commonly used shapes of SE are disk-shaped SE or square-shaped SE. In many previous cases, it was found that the processing results are better when disk-shaped SE is used rather than square-shaped SE. Line-shaped SE is used when handling thin structures or when extracting granular structures.

In this section, the results of evaluation experiments that can be used as a guide when selecting the shape of the SE and the value of *N* are presented.

Fig. S8(a) shows the results of measuring the processing effects of γ^R as the number of image rotations (*N*) increases. Under conditions where the value of *N* was varied, opening was applied to the original image of Fig. 1(a) in the main text using square-shaped SE and disk-shaped SE. The similarity between the binary images, which are the binary images of the images opened at each *N* value, and

the ground truth in Fig. 1(a) was quantitatively determined.

The root mean square error (*RMSE*) was used as a measure of similarity. The smaller the *RMSE* value, the higher the similarity between the two images being compared. Fig. S8(a) shows that, when comparing the *RMSE* values for the results of the γ^R with disk-shaped SE and the results of the γ^R with square-shaped SE, the *RMSE* values for the results of the γ^R with disk-shaped SE are smaller for all values of *N*. In other words, we can observe that the opening using the disk-shaped SE produces results that are more similar to the ground truth.

Empirically, the optimal value of *N* is considered to be 8, but it is also possible to obtain good results with smaller values. For practical purposes, it is necessary to visually check the processing results and determine the value of *N*.

The results of an experiment to find the optimal value of *N* in the γ^R using line-shaped SE are shown in Fig. S8(b). Under conditions where the value of *N* is increased, opening using line-shaped SE was applied to the original image in Fig. S7(a). Then, for each value of *N*, the obtained opening image was binarized, and the similarity between the binarized image and the ground truth was measured using *RMSE*.

Empirically, the optimal value of *N* when using line-shaped SE is considered to be 36, but good results can also be obtained with smaller values.

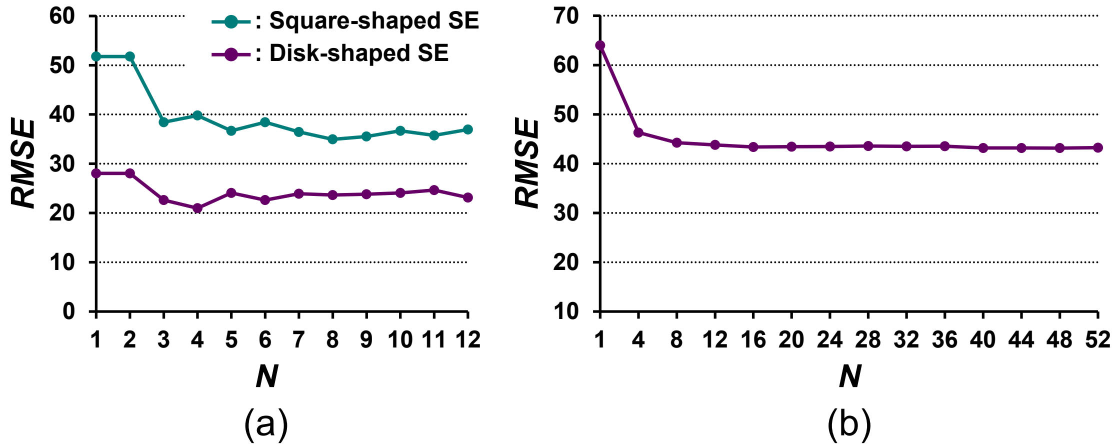

Fig. S8. (a) Results of γ^R using square-shaped SE and disk-shaped SE on the original image in Fig. 1(a) of the main text. The change in the similarity measure between the results of the γ^R and the ground truth is shown for increasing values of *N*. (b) The result of γ^R using line-shaped SE on the original image in Fig. S7(a). The change in the similarity measurement value between the result of γ^R and the ground truth is shown for increasing values of *N*.

References

[1] Schulze MA, Pearce JA. Linear combinations of morphological operators: the midrange, pseudomedian, and LOCO filters. *1993 IEEE International Conference on Acoustics, Speech, and Signal Processing*, Minneapolis, MN, USA 1993; 5: 57–60.

[2] Kimori Y. Mathematical morphology-based approach to the enhancement of morphological features in medical images. J Clin Bioinform. 2011; 1:33.

[3] Soille P, Morphological image analysis. 2nd ed. Springer; 2003.

[4] Kimori Y, Baba N, Morone N. Extended morphological processing: a practical method for automatic spot detection of biological markers from microscopic images. BMC Bioinform. 2010; 11, 373.

[5] Kimori Y, Hikino K, Nishimura M, Mano S. Quantifying morphological features of actin cytoskeletal filaments in plant cells based on mathematical morphology. J Theor Biol. 2016; 389: 123–131.

[6] Kimori Y. Morphological image processing for quantitative shape analysis of biomedical structures: effective contrast enhancement. J Synchrotron Rad. 2013; 20: 848–853.

[7] Otsu N. A threshold selection method from gray-level histograms. IEEE Trans Syst Man Cybern. 1979; 9(1): 62–66.

[8] Ljosa V, Sokolnicki KL, Carpenter AE. Annotated high-throughput microscopy image sets for validation. Nat Methods. 2012; 9(7): 637.

[9] Pettersen EF, Goddard TD, Huang CC, Couch GS, Greenblatt DM, Meng EC, Ferrin TE. UCSF Chimera—a visualization system for exploratory research and analysis. J Comput Chem. 2004; 25(13): 1605–1612.

[10] Yang F, Zhang L, Yu S, Prokhorov D, Mei X, Ling H. Feature pyramid and hierarchical boosting network for pavement crack detection. IEEE Trans Intel Transp Syst. 2020; 21(4): 1525–1535.
