# Supplementary material for: RMPJ: An ImageJ plugin for morphological information processing in biomedical images
Source: PLoS Comput Biol. 2025 Apr 16;21(4):e1012992. doi: 10.1371/journal.pcbi.1012992 (PMC12037072; doi:10.1371/journal.pcbi.1012992)
Supplement: S2 Text — (PDF) [file pcbi.1012992.s002.pdf]

RMPJ: An ImageJ plugin for morphological information processing in biomedical images

Image processing procedure using ImageJ
(Supporting Information 2)

Yoshitaka Kimori

When RMPJ is applied to actual image data, it is often used in combination with some image processing method (preprocessing or postprocessing). This section describes the specific procedures related to the image processing described in the main text.

1. Binarization of images using ImageJ functions in "Effect of the different types of SE shapes and the number of image rotations on the processing results" section

After applying γ^R to the original image shown in Fig. 1 under various conditions, the resulting images were binarized using Otsu's method. The procedure is as follows:

(1) "Image" menu > Adjust > Auto Threshold

(2) Select "Otsu" from the "Method" drop-down list

(3) Check the "White objects on black background" checkbox, and press "OK"

2. Image processing with ImageJ in the "Application to stacked image data" section

ImageJ processing to achieve the image processing flow shown in this section is as follows. The original data (mouse embryo blastocyst cells, BBBC032v1, Rivron et al. 2018) are available at the following URL.

https://bbbc.broadinstitute.org/BBBC032

- Processing step 1: The $x - y$ plane of the original data is reduced by half.

(1) "Image" menu > Scale

(2) X Scale: 0.5, Y Scale: 0.5, Interpolation: Bilinear

- Processing step 2: The image intensity bit in the original data (16-bit) is converted to 8-bit.

"Image" menu > Type > 8-bit

- Processing step 3: Gaussian blurring ($\sigma = 1$) is applied to reduce noise.

(1) "Process" menu > Filters > Gaussian Blur

(2) Sigma (Radius): 1.0

- Processing step 4: WTH^R is applied.

(1) “Plugins” menu > RMPJ

(2) Select “White top-hat” from the “Operation type” drop-down list. The shape and size of the SE and the value of the number of image rotations are determined according to the structure of each type of object to be enhanced.

- Processing step 5: To segment the WTH^R-enhanced structures, binarization is performed using Tsai’s moment-preserving thresholding method.

(1) “Image” menu > Adjust > Auto Threshold

(2) Select “Moments” from the “Method” drop-down list

(3) Check the “White objects on black background” checkbox
