# Supplementary material for: RMPJ: An ImageJ plugin for morphological information processing in biomedical images
Source: PLoS Comput Biol. 2025 Apr 16;21(4):e1012992. doi: 10.1371/journal.pcbi.1012992 (PMC12037072; doi:10.1371/journal.pcbi.1012992)
Supplement: Attachment — Submitted filename: Response_to_Reviewer.pdf [file pcbi.1012992.s005.pdf]

Response to the reviewer #2

I would like to thank you for carefully reviewing my revised manuscript and analyzing my past research and related topics for this study. In addition, I would like to thank you for your support for the development and dissemination of RMPJ. I have revised the manuscript based on your suggestions, addressing your concerns.

Comment 1: *I have a strong concern with the notion of rotation morphological processing using disk-shaped structuring element...*
To be more precise, is not clear to me which effect is implied For RMP with disk.
The global workflow involves two rotations (before and after applying morphological operation).
In addition to the morphological operation, there is therefore a smoothing effect due to the interpolations. My feeling is that the results presentged for disk are an artifact obtained from the resampling of the rotated image, even if I could not find a way to demonstrate it...
Also, while linear interpolation is the most classical choice, other methods could be chosen, possibly leading to different results.
Anyway, relying on such an effect for presenting the software, or more generally to use as basis the differences in the discretisations of a disk, seems to me highly discutable.
Moreover it may lead to controversies about the general validity of the RMP method/framework, that would be detrimental to the presented software.

Author response: I understand your concerns regarding the use of disk-shaped SE in RMP. To address this, I have conducted a number of experiments. Through the results of these experiments, I hope to clarify the effects of RMP using disk-shaped SE and the reasons for using disk-shaped SE.

To begin with, in Section 1 “Discrete representation of disk-shaped SE and opening operation using square-shaped SE and disk-shaped SE”, I demonstrate the disk shapes used in RMPJ, which are represented discretely. In addition, I compare the results of opening using square-shaped SE and disk-shaped SE.

This experiment shows **that disk-shaped SE is more useful than square-shaped SE and that RMP using disk-shaped SE shows the highest performance in terms of preserving the shape of the object being analyzed in image processing processes such as noise removal.**

Furthermore, I believe that the influence of interpolating pixel values using rotation transformation is a crucial factor that should be considered in RMP. I designed RMPJ with extreme care to minimize this effect as much as possible. For details, please refer to Section 2, “The influence of interpolation processing during rotation transformation and approach to correcting it”. In this section, I have designed the RMP framework transparent by explicitly showing the intermediate images obtained during the procedure of RMP.

As shown in Figs. R5–R7, the results of the opening operation using a disk-shaped SE are not affected by the interpolation processing (smoothing) owing to rotation transformation.

In addition, RMP operates to combine multiple rotated images. The intensity values of the combined image are corrected such that they are consistent with the concept of traditional morphological operations. The details are shown in Fig. R7.

From these results, we can conclude that it is reasonable to think that the outcome of opening operation based on RMP depends only on the efficacy of opening operation.

Moreover, regarding your next comment: "*while linear interpolation is the most classical choice, other methods could be chosen, possibly leading to different results*", I believe that opinion is completely correct. The results will differ depending on the interpolation method used. Therefore, when implementing RMP, I have been considering multiple interpolation methods. As shown in Figs. R5–R7, I outputted intermediate images during the processing process using RMP and displayed them in pseudo-color images to analyze the effect of interpolation on the structures in the images.

Consequently, I concluded that the bilinear method was the most suitable from both the perspective of preserving structure and computational time cost and adopted it. (In the experiment shown in Fig. R7, when the bilinear method was used, the range over which the intensity values changed owing to the influence of the interpolation process was approximately 2 pixels. With other methods, this range is even wider, and it is considered that this may have a negative impact on the preservation of the image structure).

Comment 2: *However, I am strongly convinced that the RMP method is very useful and well adapted for non-disk structuring elements.*
I strongly encourage to revise the manuscript, by putting more emphasis on square and linear SE, and removing the presentation of RMP based on disks.

Author response: I believe that the proposal to consider the application of RMP with a focus on the use of nondisk SE is extremely valuable because it makes it easier to emphasize the uniqueness of RMP theory.

However, this paper introduces a software called RMPJ. I have developed RMPJ to be a useful tool for gaining new insights into the life sciences. I believe that the usability of RMPJ is an important factor that must be ensured first to meet the needs of many users.

If the disk-shaped SE is removed from RMPJ, then RMPJ will lose its usefulness. For usability, I would like to keep the disk-shaped SE as one of the options for SE.

When implementing RMP, I set myself the following two goals:

(1) To preserve the structure of the object being analyzed as much as possible during the image processing process.
(2) To improve the usability of the RMPJ software.

During the process of developing RMPJ, I processed various image data, but it was occasionally difficult to achieve these objectives with square-shaped SE and linear-shaped SE. Furthermore, **to achieve these goals, it was necessary to use disk-shaped SE.**

For example, in the RMP opening using a disk-shaped SE ($N = 8$), unwanted structures such as noise are removed, but the structure of the target for analysis is preserved. In terms of preserving the structure of the target for analysis, the RMP using a disk-shaped SE outperforms the RMP using a square-shaped SE.

In addition, in general, when performing morphological analysis, it can be difficult for users to select the optimal shape of the SE. Therefore, an SE that is more practical and versatile is required. The disk-shaped SE accomplishes this task.

The usefulness of disk-shaped SE is demonstrated through the experiments in Sections 1 and 3, "Comparison of the results of opening operations for square-shaped SE and disk-shaped SE".

To ensure the usability of RMPJ, I would like to enable users to select the disk-shaped SE.

Please see the following experimental results and support the RMPJ with the integrated disk-shaped SE.

Section 1. Discrete representation of disk-shaped SE and opening operation using square-shaped SE and disk-shaped SE

Here, I would like to clarify the shapes of the various sizes of disk-shaped SE used in RMPJ. RMPJ uses the ImageJ function to draw disk shapes. Figure. R1 shows disks drawn discretely. The smaller the size, the greater the difference from the analog representation of a circle. In processing, SE sizes of 11 pix or less are often used.

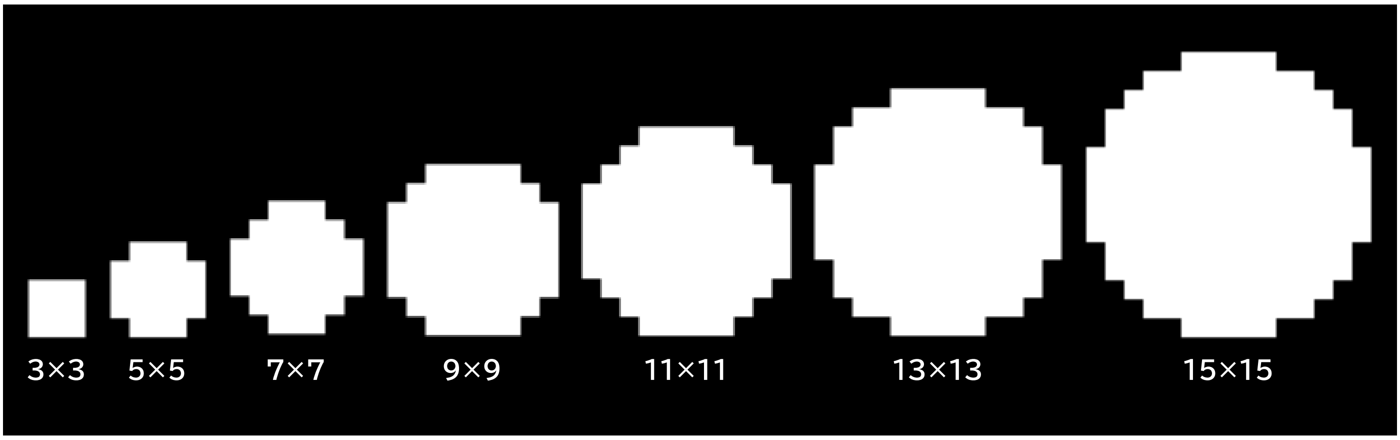

Fig. R1: Discrete disk shapes, with sizes ranging from 3×3 pixels to 15×15 pixels.

As shown in Fig. R1, the shape of a disk cannot be expressed as a continuous "circle." Therefore, similar to the case of using a square-shaped SE, in many cases, the aforementioned goal (1) cannot be achieved using traditional morphological operations.

Even in the case of a disk-shaped SE, it is most useful in practical terms when used in RMP.

In the following, the usefulness of RMP with disk-shaped SE is demonstrated through some practical examples.

First, we consider the opening using the square-shaped SE for the square objects (Fig. R2(a)). In the opening operation, the region where the SE fits is preserved and the area where the SE does not fit is removed. Fig. R2(a) shows the original image. The O_2 shown in this image was created by rotating O_1 45° clockwise. Fig. R2(b) shows the result of the traditional opening using the square-shaped SE (the same as the $N = 1$ condition in RMP). Because the square-shaped SE fits to all regions of O_1, its shape is preserved. By contrast, in O_2, the regions where the square-shaped SE does not fit have been removed. Fig. R2(c) shows the removed regions (i.e., the difference between the original image and the opened image in the O_1 and O_2 regions) in magenta.

Here, O_1 and O_2 are considered to be objects of the same shape, differing only in orientation; therefore, even if the opening operation is applied to the original image, it is desirable that the structure of O_2 is preserved as it is.

However, this processing cannot be achieved using traditional morphological operations.

Therefore, RMP opening with $N = 8$ was applied to the original image. The result is shown in Fig. R2(d). By rotating the image, the square-shaped SE fits the entire region of O_2; therefore, the overall shape is preserved. In the O_1 and O_2 regions, the residual between the original image and the result of RMP opening is zero (Fig. R2(e)). In other words, as long as RMP is used, the square-shaped SE may fit the entire region of any square object, regardless of its orientation.

This is an advantage of RMP that is not available in traditional mathematical morphology.

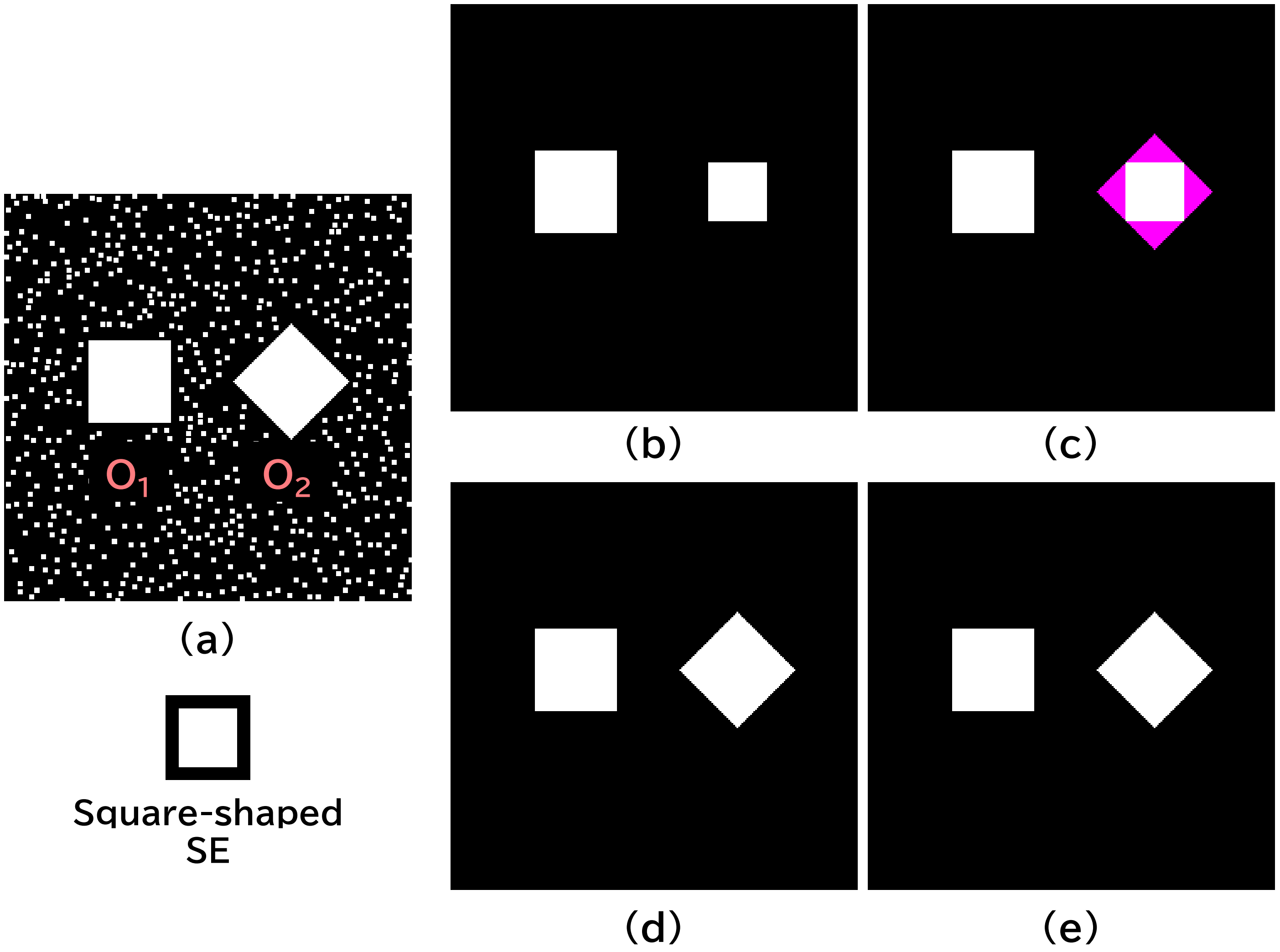

Fig. R2: Opening using a square-shaped SE. The RMP opening ($N = 8$) removes noise while preserving the structure of the target objects O_1 and O_2.

Next, we consider the opening of disk-shaped objects using disk-shaped SE.
If we could perform the traditional opening operation using disk-shaped SE in the continuous analog domain, then the entire region of the disk-shaped object would be perfectly preserved (in this case, the concept of RMP would not be necessary). However, we are handling digital images. A significant difference exists between the discrete shape representation of a disk and its continuous representation.

Fig. R3(a) (original image) shows two disk-shaped objects that were represented discretely. O_2 was created by rotating O_1 clockwise by 45° and is considered to be the same shape as O1. The positions ① and ② shown at the top of O1 correspond to the positions ①'and ②' in O_2, respectively.

Fig. R3(b) shows the result of applying the traditional opening ($N = 1$ under RMP conditions) using disk-shaped SE to the original image. Because this is an opening using a disk-shaped SE for disk-shaped objects, the SE is expected to fit the entire regions of O_1 and O_2 and preserve their shapes. However, considering the results of this opening, we can observe that the shapes of O_1 and O_2 differ from the original image. In the regions of O_1 and O_2, the difference or the regions removed by the opening, are shown in magenta in Fig. R3(c).

In the case shown in Fig. 2, a region has also been removed from the O_1 object. This means that owing to the difference in the way the discrete disk shape of O_1 and SE is represented, some regions exist where this SE does not fit in the entire region of O_1. In contrast to the case of processing a square-shaped SE for a square (or rectangular) object, we can observe that, in the opening process using a disk-shaped SE, some regions exist where the disk-shaped SE does not fit owing to the representation of the discrete disk shape, as well as the orientation of the SE and object.

Fig. R3(d) shows the result of applying RMP opening with $N = 8$ to the original image. Compared to the result of the traditional opening (Fig. R3(b)), we can observe that the shape of the disk-shaped object in the original image has been preserved. In addition, in the regions of O_1 and O_2, the processing results are nearly identical to the original image (Fig. R3(e)). This is because the disk-shaped SE fits into these regions by rotating O_1 and O_2.

From these results, we can conclude that even if a disk-shaped SE is used, the results of the traditional opening and RMP opening will not be the same.

If the size of the object to be processed is sufficiently large compared to the SE, the SE may fit the entire region of the object to be processed, even if it is a traditional opening. However, in actual image processing, the size of the SE is slightly smaller than the size of the object to be processed. Therefore, cases similar to as shown in Fig. R3 can occur quite often.

From the perspective of achieving the aforementioned goal (1), even when using a disk-shaped SE, we can conclude that RMP opening ($N = 8$) is suitable.

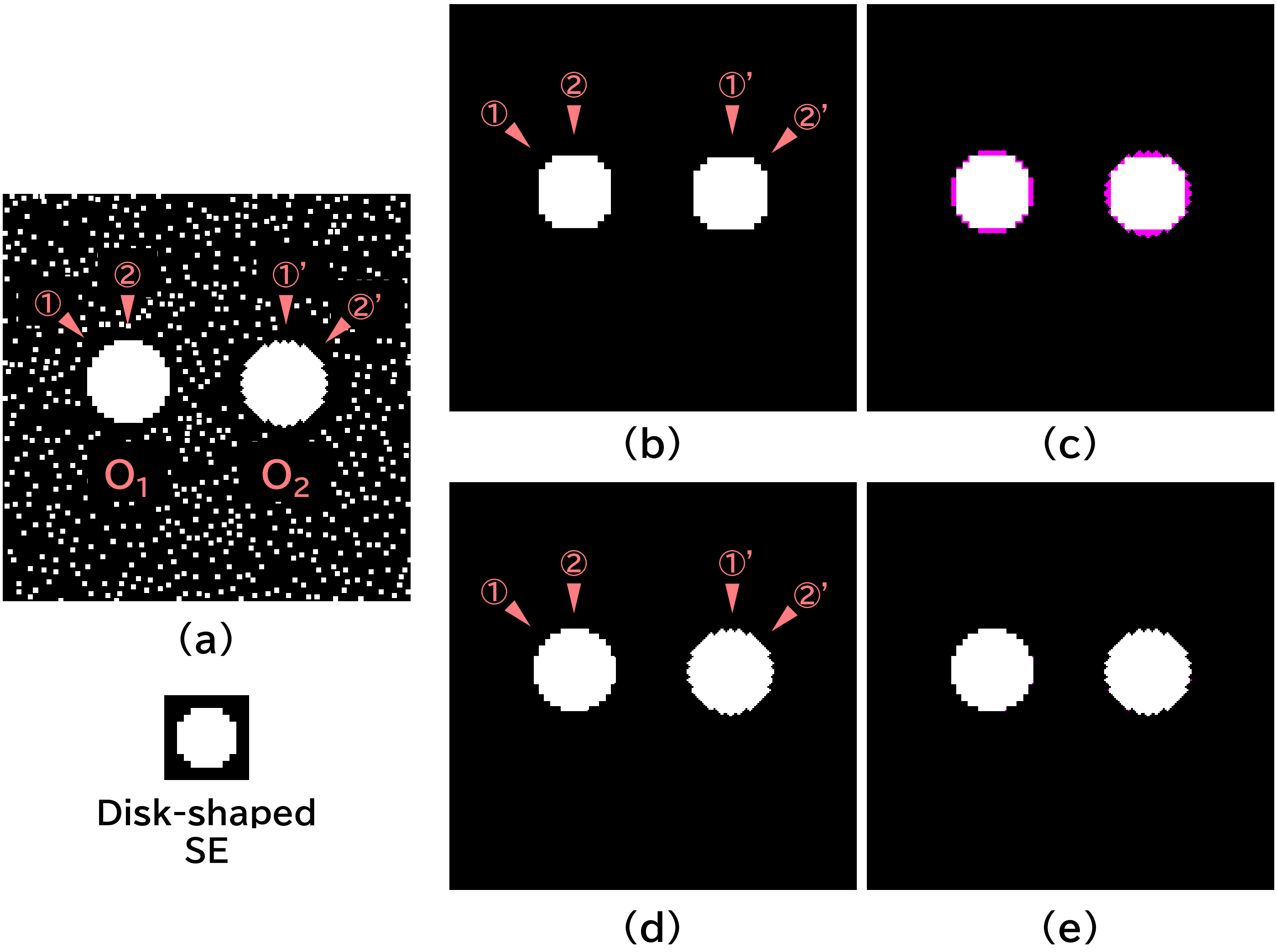

Fig. R3: Traditional opening using the disk-shaped SE discretely destroys the structure of the target objects. By contrast, the RMP opening ($N = 8$) can preserve the structure of the target objects even when the same SE is used.

In certain cases, the disk-shaped SE can be replaced with a square-shaped SE. Here, we attempt applying the opening using the square-shaped SE to the original image, Fig. R4(a) (the same image as Fig. R3(a)). The width of the square-shaped SE is the same as the disk-shaped SE in Fig. R3. Fig. R4(b) shows the result of applying the traditional opening ($N = 1$ under the RMP conditions), and the difference in the regions of O_1 and O_2 from the original image is shown in Fig. R4(c). From the result, we can observe that there is more residual region than the result using disk-shaped SE (Figure R3(c)). **Even if the width of the square-shaped SE and disk-shaped SE is the same size, the square-shaped SE has four corners; therefore, the area is larger than the disk-shaped SE. Consequently, the region that fits is more limited than with the disk-shaped SE.**

Fig. R4(d) shows the results of RMP opening for $N = 8$. When the disk-shaped objects were rotated 45°, we can observe that the square-shaped SE was able to fit inside the region of the objects.

Furthermore, the value of N was increased. Fig. R4(f) shows the results of RMP opening for $\boldsymbol{N} = \mathbf{32}$. This result was equivalent to the result of RMP opening for $\boldsymbol{N} = \mathbf{8}$ using the disk-shaped SE.

From this, we can conclude that the disk-shaped SE can be substituted for the square-shaped SE when processing this original image. However, because the value of N is large, the computational cost is extremely high. **Therefore, we can conclude that, in practical terms, processing using the disk-shaped SE is more useful than using the square-shaped SE.**

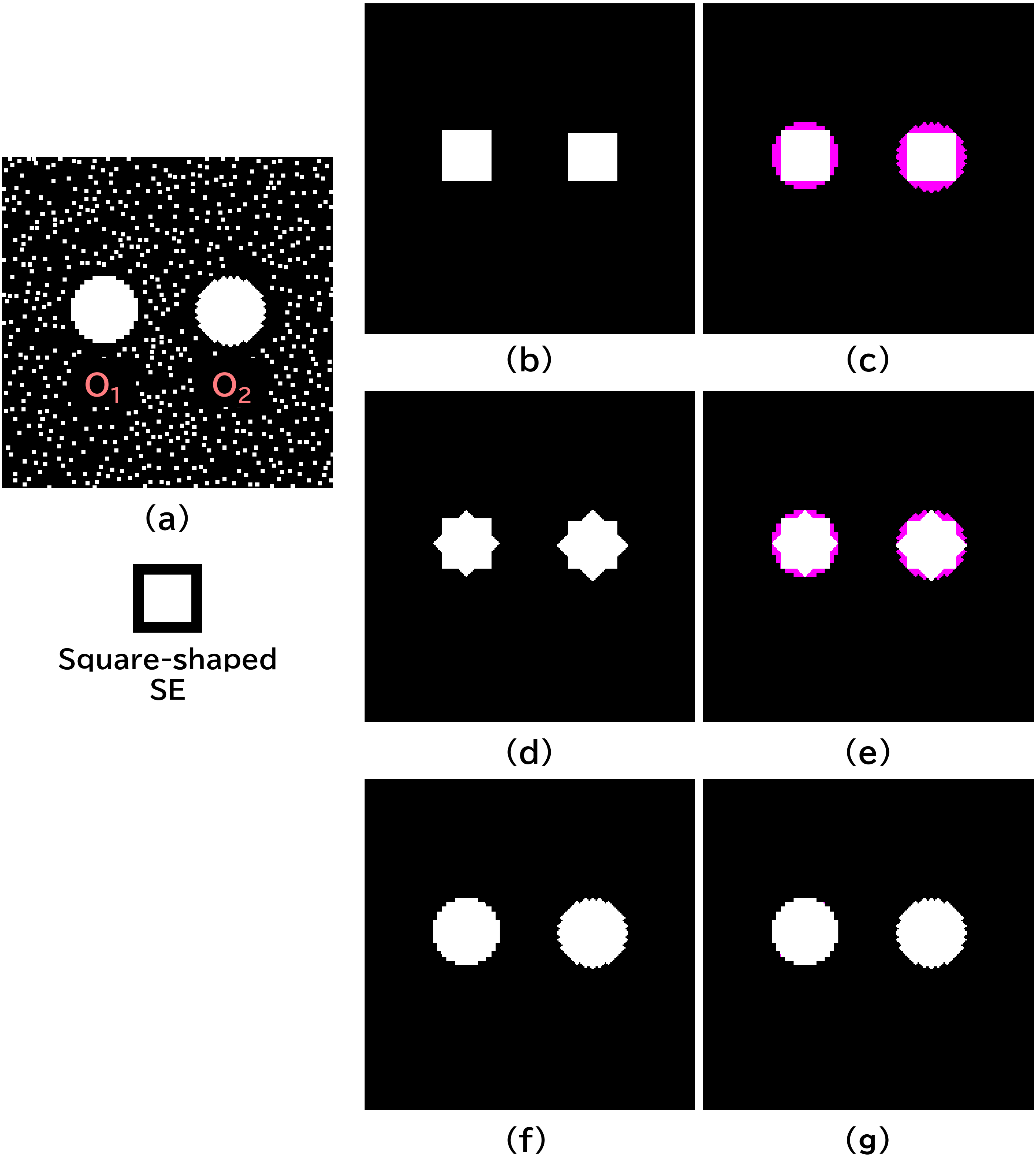

Fig. R4: In RMP, in certain cases, a disk-shaped SE can be substituted with a square-shaped SE. However, it requires more computational cost to obtain the same result as when using a disk-shaped SE.

Section 2. Influence of interpolation processing during rotation transformation and approach to correcting it

In this section, I will describe the effect of interpolation operations on the rotation transformation of RMP. First, I will apply the opening process to the image on the left of Fig. R5(a). Some patterns of black and white line structures are arranged. The width of the line structures is 3 pixels, 5 pixels, and 7 pixels. I will perform RMP opening using disk-shaped SE with a width of 9 pixels on this image. The right image in Fig. R5(a) is a pseudo-color image of the left image. We can visualize pixel values that are not visible. For example, pixels with a pixel value of 1 are represented in blue.

Fig. R5(b) shows the results of the traditional opening (RMP opening under the condition of $N = 1$, **that is, no rotation transformation is applied**). Fig. R5(c) shows the results of the RMP opening under the condition of $N = 8$. Comparing these results, we can observe that they are the same.

If there are any artifacts in the RMP opening result image owing to rotation transformation, they should appear as changes in intensity values in the image shown in Fig. R5(c). However, we can observe that there are no such artifacts in the pseudo-color image.

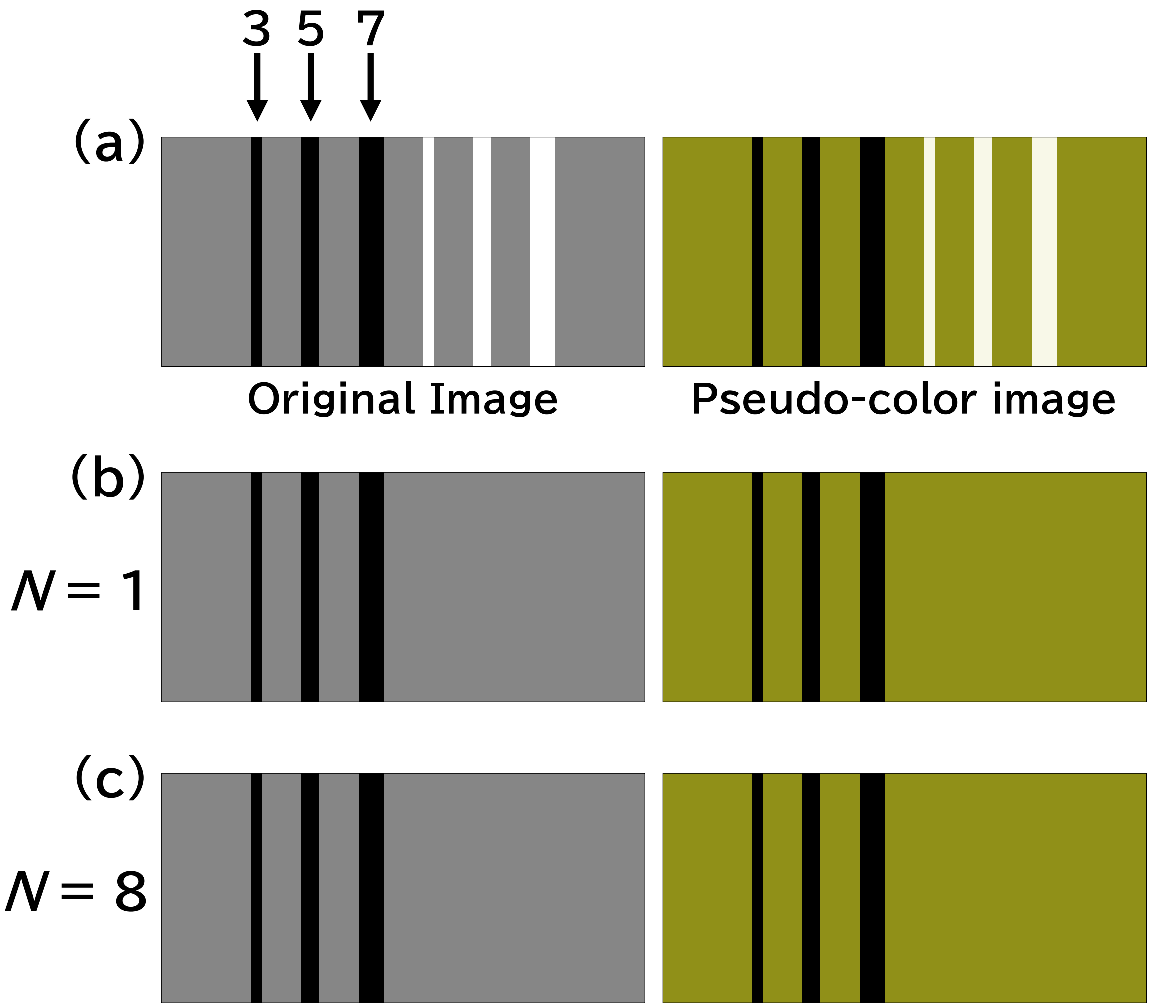

Fig. R5: Results of opening for the image (a) are the same for both the traditional processing (b) and RMP (c). The widths of the linear structure patterns in the image in (a) are 3, 5, and 7 pixels. The opening was performed using a disk-shaped SE with a width of 9 pixels. It is not considered that an artifact exists where the white linear structure patterns disappear in the rotation conversion of the RMP opening. Therefore, it is evident that the white linear structure patterns were removed by the function of the opening.

Next, we will attempt to visualize them to understand the impact of interpolation operations in rotational transformation. Fig. R6 shows images rotated by the rotation angles for the case of $N = 8$ in RMP. The images were rotated twice, clockwise and counterclockwise, at each angle. (Only rotation transformations were performed, and no morphological processing was performed).

When comparing a pseudo-color image with a rotation angle of 0° with a pseudo-color image that has been interpolated using rotational transformation (for example, an image with a rotation angle of 22.5°), we can observe that the intensity patterns of the linear structures differ from those of the pseudo-color image with a rotation angle of 0°. In the region of the edge of the linear structure patterns, intensity values change owing to interpolation operations within a range of two pixels.

However, the degree of this influence is small (the range of pixels suffering from the influence is 2 pixels), and it is not enough to make the object being analyzed disappear. The patterns of the white linear structures have not disappeared. Therefore, we can conclude that the removal of the patterns of white linear structures is the result of the contribution of only the opening operation in RMP.

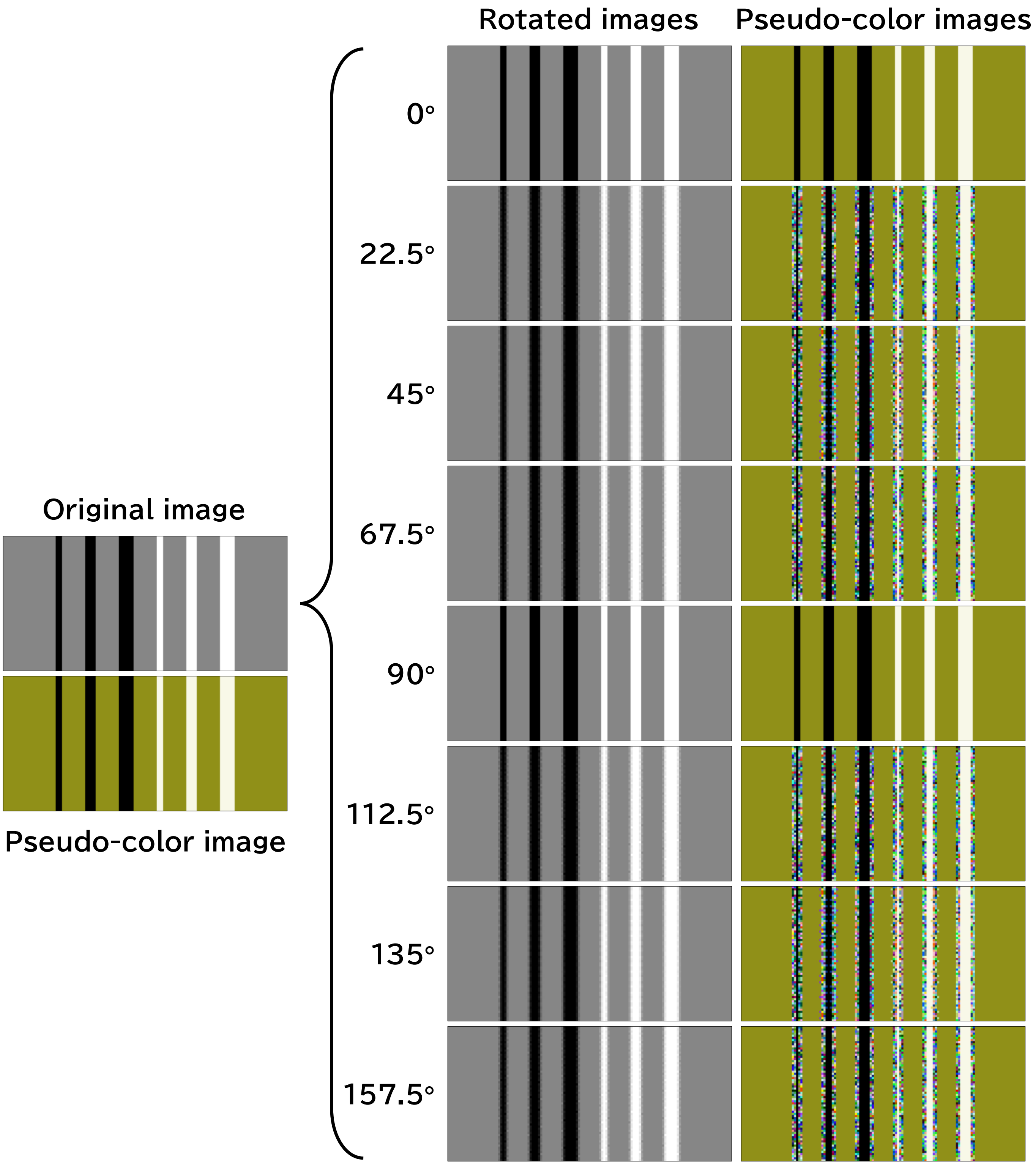

Fig. R6: Rotated images after two rotations, clockwise and counterclockwise. No artifacts were observed that would remove the patterns of the white linear structures.

However, rotation transforms intensity values that differ from the original image (even if the influence is small).

Therefore, I have already incorporated a mechanism to correct the negative effects of this rotation transformation into RMPJ. Figure R7 shows the process of applying RMP opening to the original image with $N = 8$. The images of the opening processing results for each rotation angle are shown (for example, the image with the "22.5°" display is the

image after the original image has been rotated 22.5° clockwise, the opening operation has been applied and then rotated 22.5° counterclockwise). **If these opened images are compared with the rotated images in Fig. R6, it is evident that the patterns of the white linear structures were removed by the opening function**.

In RMP opening, these opened images are combined using maximum operation. However, when comparing the intensity values of the pixels in the combined image with the corresponding pixels in the original image, certain cases exist where pixels with higher intensity values than the original image appear in the combined image. In traditional opening operation, when comparing the intensity values of the corresponding pixels of the original image and the opened image, the intensity value of the opened image will never exceed the intensity value of the original image.

Therefore, RMPJ compares the corresponding pixels of the combined image and the original image, and if the pixel value of the combined image is larger than that of the original image, it performs a process to replace the pixel value of the combined image with that of the original image. (in the case of closing, if a smaller intensity value than the original image is produced in the integrated image, it is replaced with the value of the original image). **This operation ensures consistency with the traditional morphological operation. This RMP framework allows to output images that preserve the desired structure while removing unnecessary structures.**

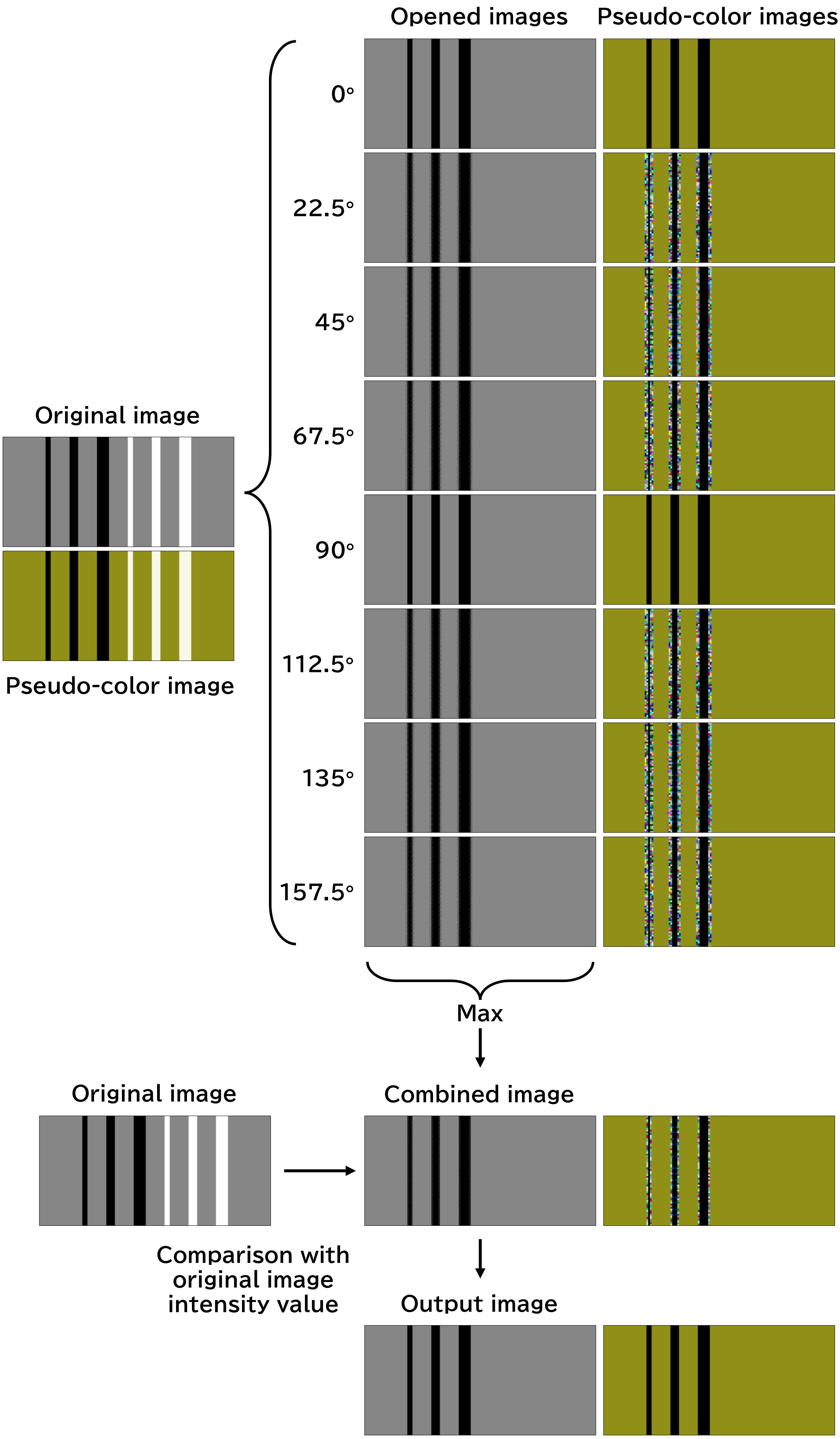

Fig. R7: Correction of the intensity values of the combined image generated by the maximum value operation in the RMP opening.

Section 3. Comparison of the results of opening operations for square-shaped SE and disk-shaped SE

This section presents examples of applying RMP to images with more complex intensity distributions than the previous images and validates the usefulness of disk-shaped SE.

Fig. R8(a) (left) shows the original image (crystalline_0107.tif, which was obtained from the Describable Textures Dataset (DTD) (https://www.robots.ox.ac.uk/~vgg/data/dtd/) and converted to grayscale). Fig. R8(a) (right) shows the image with the original image expressed in pseudo color. Opening was applied to this original image using square-shaped SE and disk-shaped SE. The results of RMP opening with square-shaped SE are shown in Fig. R8(b) ($N = 1$) and Fig. R8(c) ($N = 8$). The results of RMP opening with disk-shaped SE are shown in Fig. R8(d) ($N = 1$) and Fig. R8(e) ($N = 8$).

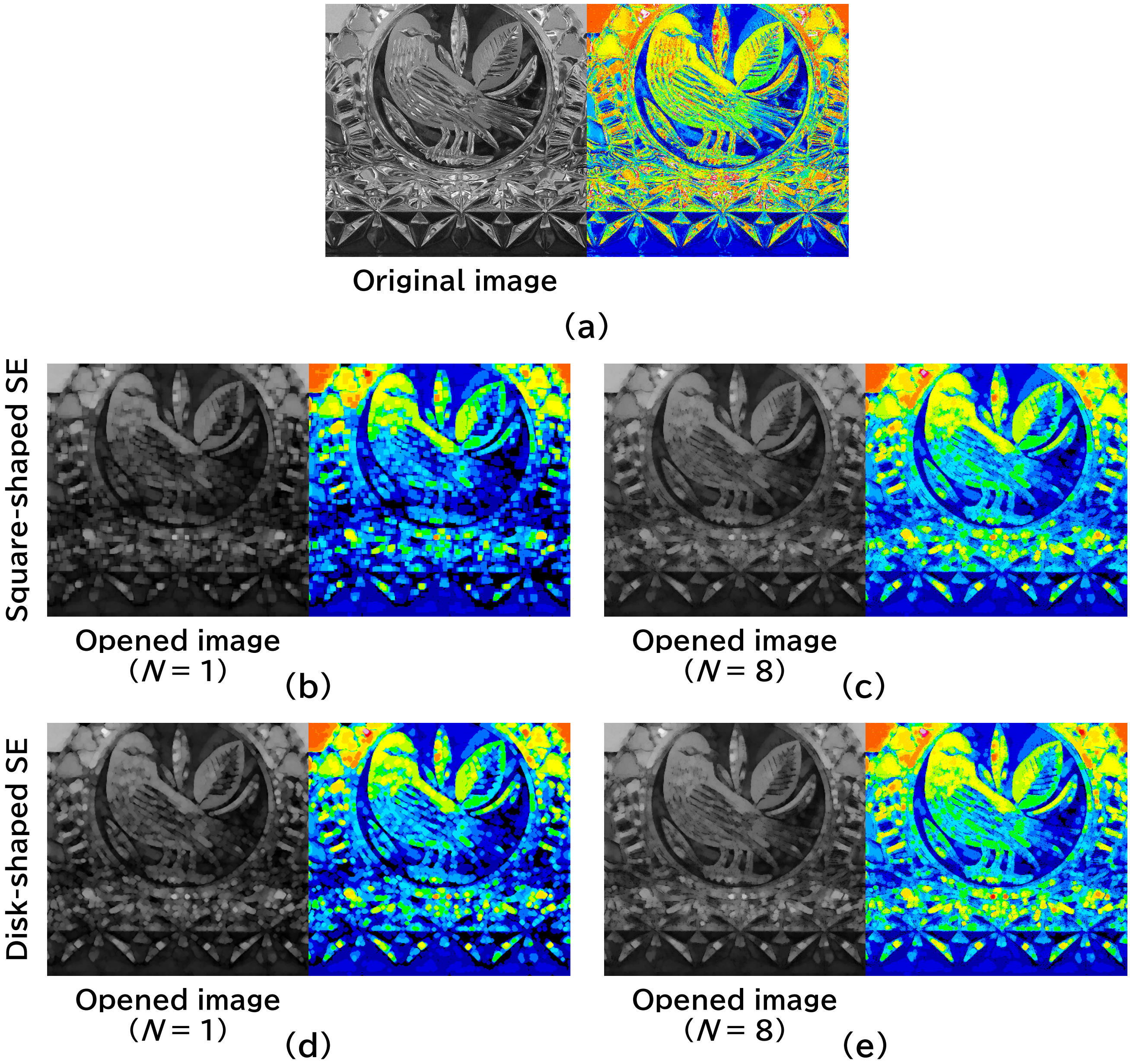

Fig. R8: Results of opening operations using square SE and disk-shaped SE

Let us closely observe the results of these opening processes. Fig. R9 shows the results of opening using disk-shaped SE ($N = 1$ and $N = 8$) (see the areas surrounded by the dotted lines). **The results of RMP opening (N = 8) show that many of the detailed structures in the original image are preserved well compared to the results of RMP opening (N = 1).** These results show the same trend as the experimental results shown in Fig. R3.

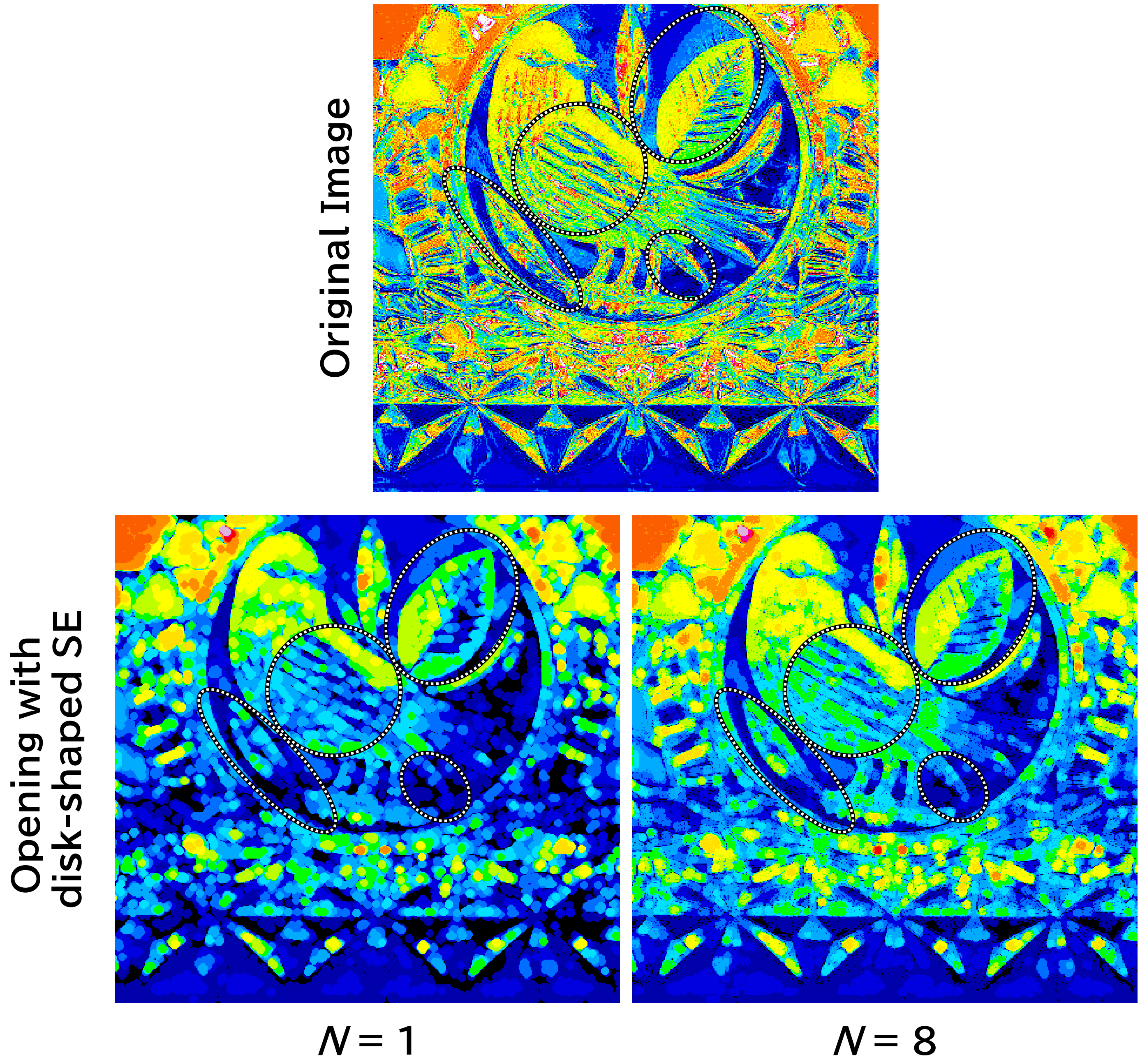

Fig. R9: Results of opening using disk-shaped SE. RMP opening ($N = 8$) preserves the detailed structures in the image.

Fig. R10 shows the results of opening using square-shaped SE and disk-shaped SE. In both cases, $N = 8$. If the areas surrounded by the dotted lines are examined, **we can observe that the results using disk-shaped SE more efficiently preserve the structural details.** With square-shaped SE, certain areas exist where the structure disappears.

The results indicate that in this case the RMP opening using the disk-shaped SE (N = 8) can achieve the aforementioned goal (1).

These results indicate that disk-shaped SE is a useful SE for practical purposes, and this SE is essential for RMPJ to meet the needs of more users.

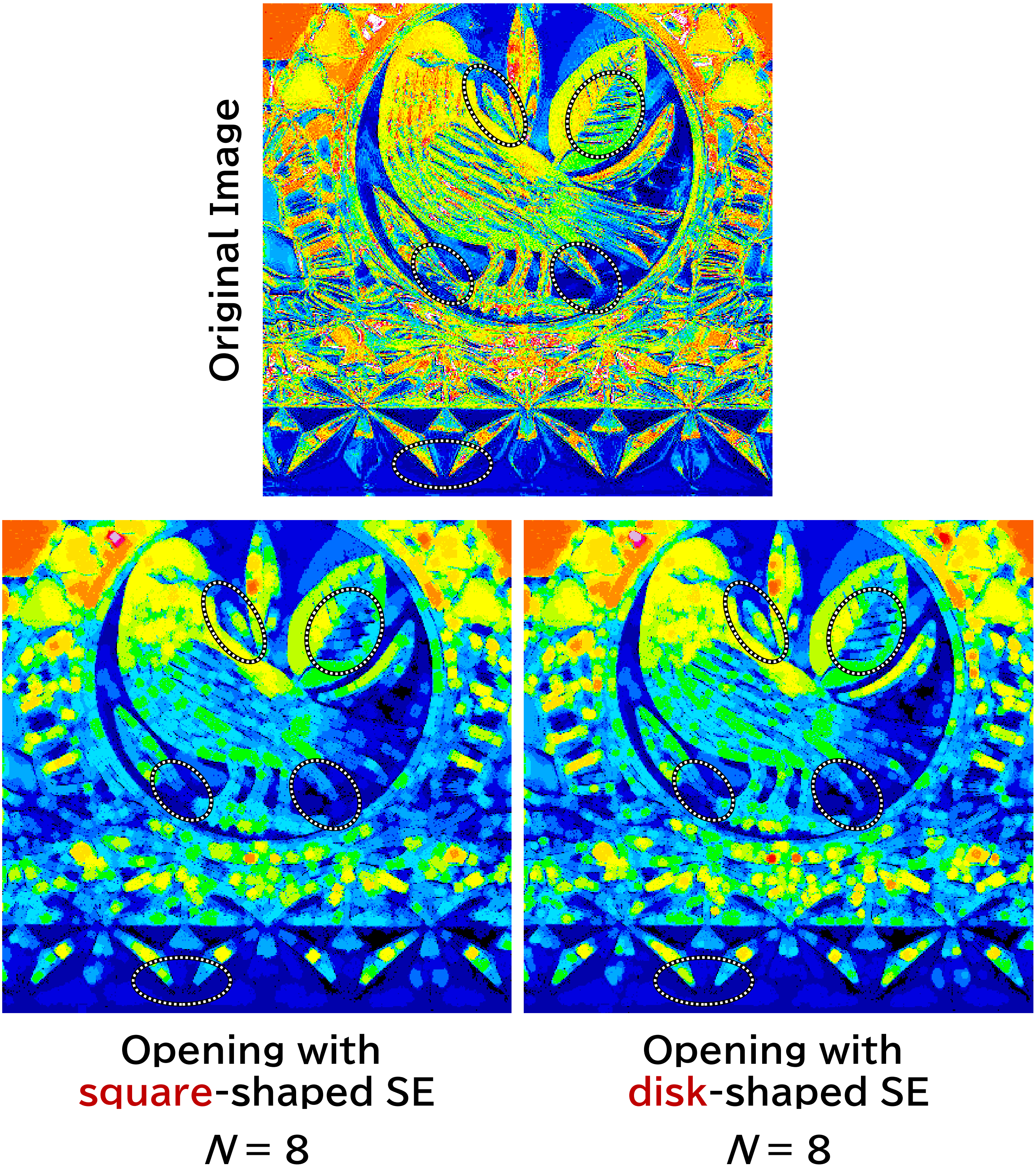

Fig. 10: Results of the opening using square-shaped SE and disk-shaped SE. RMP opening using square-shaped SE does not sufficiently preserve the structures in the image.

Comment 3: *I also feel there is room for further investigations on the links between RMP, path-opening, and related methods... Even if larger comparison is out of the scope of the manuscript, it would be beneficial to add few references to link to related works (for example in the introduction).*
Author response: Thank you for your comments on related research. In the "Availability and Future Directions" section, I have cited the references that you provided. In addition, I plan to conduct a study of the comparison between RMP opening and path-opening in the future (pp. 18–19, lines 320–325).

Comment 4: *more minor point, I would advocate of avoiding the "iterations" term. Using the term "iteration" may lead the reader that the algorithms updates its internal state at each iteration, such that each iteration depends on the previous one. Typical examples are k-means or gradient descent. In this case, it seems the term 'rotation angle' could be used more adequately?*
Author response: Thank you for highlighting this. It is true that if the term "iteration" is used, it may be understood as performing a further rotation transformation on the rotated image.

However, because "rotation angle" is defined as θ, I would prefer to change N to "number of image rotations" (p. 10, line 167).

Comment 5: *I am sorry to say that this does not help understand the principles that makes the RMP framework seem to work... As discussed previsouly, I would "incriminate" interpolation artifacts.*
Author response: In the experiments shown in Section 2 (Figs. R5-R7), we can observe that no artifacts caused by interpolation operations that exceed the function of opening appeared.

The RMP framework is definitely based on the principles of mathematical morphology and possesses the efficacy of opening.

Comment 6: *I have the impression that a classical top-hat with a disk (not rotational) would have the same result. This enforce me in the conviction to avoid putting the emphasis in RMP with disk.*
Author response: Because the top-hat transform is the difference between the original image and the opening image, the quality of the opening result will affect the quality of the resulting image. As shown in the experiments in Section 3, in the case of opening using a square-shaped SE or the traditional opening using a disk-shaped SE, certain regions exist where the structure of the original image is destroyed; therefore, a concern exists that these regions will be extracted as artifacts. When using a square-shaped SE, and even when using the same disk-shaped SE, the results of the top-hat transform will differ between the $N = 1$ and $N = 8$ conditions.

RMP top-hat transforms ($N = 1$ and $N = 8$ conditions) using square-shape SE and disk-shaped SE are applied to the original image in Fig. R11(a) (this image is the original image in Fig. R3 of the previous response letter). The purpose of this processing is to accurately extract regions of the same width from the filament structures in the original image.

Figs. R11(b) and R11(c) show the results of the RMP top-hat transform using square-shaped SE. These are the results for $N = 1$ and $N = 8$, respectively. In both cases, the image on the left is the result of the top-hat transform, and the image on the right is the binary image of the result. Considering these binary images, relatively large artifacts exist in R11(b), and the shape of the square-shaped SE is reflected in R11(c). Furthermore, the outline of the extracted filament is rough and not smooth, with the presence of small isolated fragments.

Figs. R11(d) and R11(e) show the results of RMP top-hat transformation using disk-shaped SE. The conditions are $N = 1$ and $N = 8$, respectively. We can observe that certain artifacts exist in the binary image of R11(d). During the opening process, regions that the SE could not fit were extracted as artifacts.

In contrast to the results of the other top-hat, the results of R11(e) show that width of filament regions of nearly uniform width have been extracted, with no artifacts. **Even from this example of image processing, we can observe that using disk-shaped SE is effective in RMP.**

In image measurement, the top-hat transformed image is binarized and the binarized image is measured. Therefore, avoiding the occurrence of artifacts through morphological processing is essential. Therefore, the use of disk-shaped SE becomes more important.

In the development of the RMP to date, I have ensured preventing the appearance of pixel errors (i.e. artifacts) (even if they are pixels with intensity values that cannot be seen with the naked eye).

From this experience, I believe that the disk-shaped SE is a safe SE with a low probability of the appearance of such artifacts and that the use of this SE is essential to use the RMPJ more effectively.

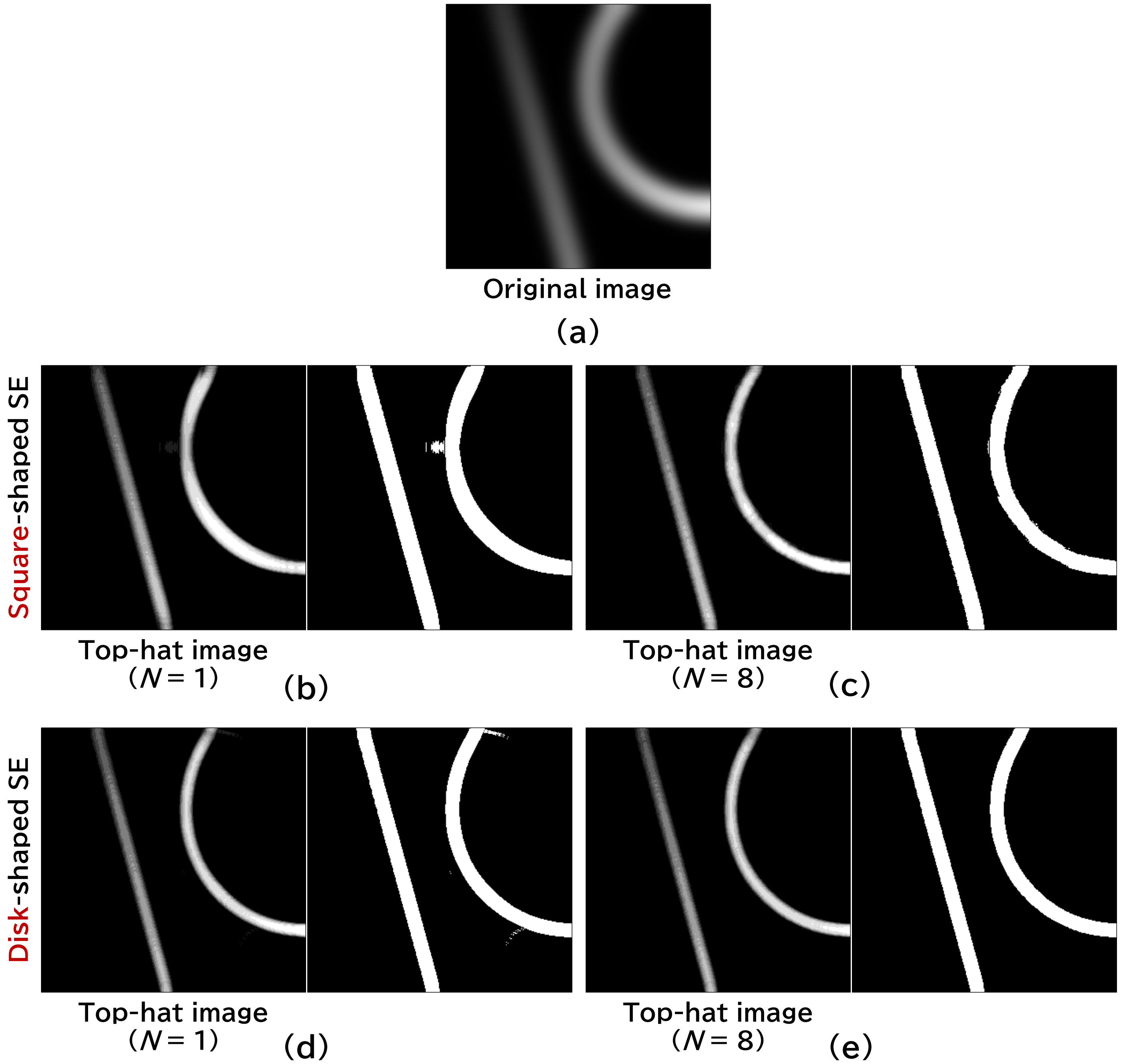

Fig. R11: Filament regions can be extracted most successfully using the RMP top-hat transform with $N = 8$ conditions using disk-shaped SE.

Comment 7: *L59: Some authors include the terms "resp." within parens to make explicit that the term within parens is the alternative case as the one in plain text. https://math.stackexchange.com/questions/3826526/what-this-resp-means*

Author response: Thank you for highlighting this. I have corrected the relevant sentence using the term "resp." (p. 5, lines 76–78; p.9, lines 146–149).

Comment 8: *L66: This paragraph is ambiguous and discutable. If the SE is a (contiunous) disk, it *is* rotation invariant, and its discretisation will remain the same.*

*However, What I understand from the paragraph, is that the *discretisation* of the SE is not invariant to rotation. At*

least add "discretized" before "shape" would be enough to clarify.
Author response: The term “discretized” has been added. The addition of this term has made the meaning of the sentence clearer (p. 5, line 84).

Comment 9: *L86: remove "iteratively". while the method uses a for-loop, this is not an iterative algorithm that updates its state after each iteration.*
Author response: The term “iteratively” has been deleted from the relevant sentence (p. 6, lines 102–104).

Comment 10: *L137: I would rephrase as "The RMPJ plugins allows to setup four input options:"*
Author response: The relevant sentence has been corrected (p. 9, line 156).

Comment 11: *L148: I would replace by "the number of image rotations to consider" (one may think that rotations are applied in successions)*
Author response: As this text is displayed in the RMPJ GUI, it needs to be as short as possible. Therefore, I have changed it to “Number of image rotations” (p. 10, line 167).

Comment 12: *L186: I am still convinced that for this example it would be more appropriate to use line structuring elements...*
Author response: I will attempt processing using a line-shaped SE. The top row of Fig. R12(a) shows the original image (Fig. 2 in the main text), and the bottom row shows the GT. The top row of Fig. 12(b) shows the result of RMP ($N = 8$) using a disk-shaped SE (size: 7 pixels), and the bottom row shows the binary image of the same. Fig. R12(c) shows the results of RMP opening ($N = 36$) using line-shaped SE. The results for SE lengths of 7 pixels, the same size as the disk-shaped SE, show that a lot of noise remains. This is because the width of the line-shaped SE is 1 pixel; therefore, it is easy to fit the noise structure.

The results of increasing the length of the SE to 15 pixels are also shown. Even when the length of the SE is increased to 15 pixels, the background noise is not sufficiently removed. If the length of the SE is increased further, the mesh structure begins to break down.

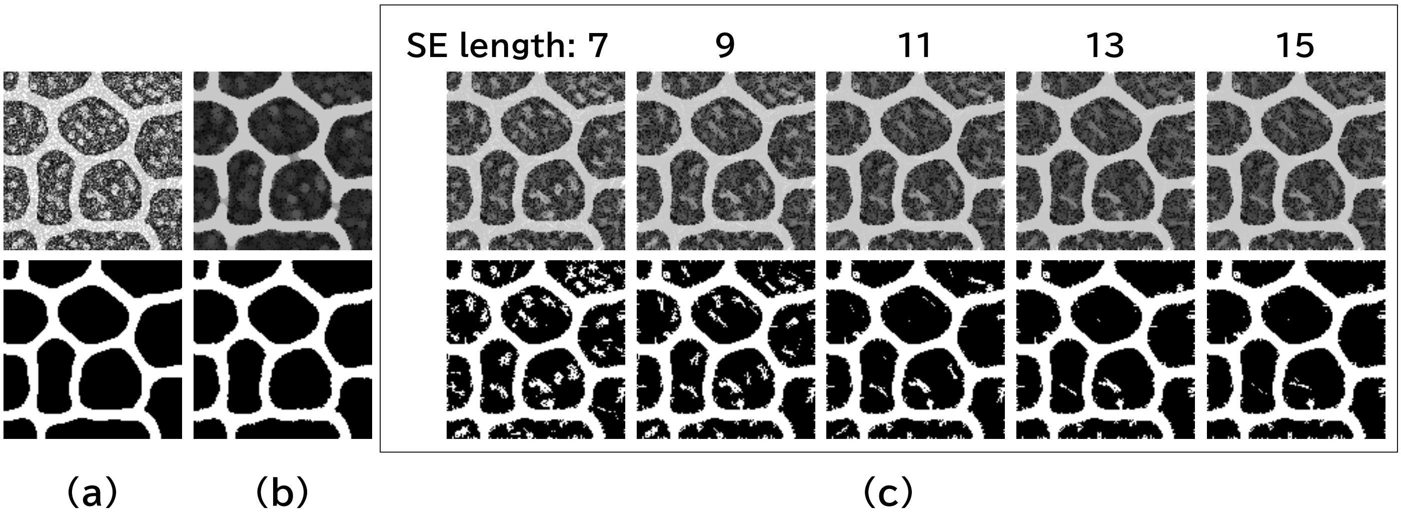

Fig. R12: Extraction of mesh structure using RMP opening with line-shaped SE. The noise was not sufficiently removed in the process using line-shaped SE.

A grayscale image can be considered to have a 3D structure, with the intensity values corresponding to the height. Morphological processing for grayscale images must consider the way that SE fits this 3D structure. When selecting SE, it is not sufficient to consider only the 2D size of the objects in the image.

To understand this, a simple image (a binary image) similar to Fig. R13 (left) is created, and RMP opening using line-shaped SE is applied to it. Noise removal can be achieved successfully even when using line-shaped SE (the processing result is shown on the left of Fig. R13). In this case, it is sufficient to consider the size of the noise (the region in 2D).

In general, it is difficult to grasp the 3D intensity distribution of a grayscale image; therefore, we consider grayscale images simple, as shown in this example. However, this approach often does not select the optimal SE.

In the image in Fig. R12(a), the line-shaped SE is easy to fit to the local region of noise, which is composed of a 3D intensity distribution; therefore, the use of this SE is not appropriate for this image. The selection of the optimal SE for this image is performed according to the following ideas.

(a) To avoid fitting to local regions of noise, SE with a wider width than line-shaped SE, that is, square-shaped SE and disk-shaped SE, are candidates.

(b) Preserving the shape of the region of the mesh structure is necessary while removing noise. Therefore, it is optimal to select disk-shaped SE, which is considered to fit the region well, from the candidates in (a).

In addition, **using disk-shaped SE, RMP opening under the condition of $N = 8$ achieved the aforementioned goal (1).** The results are presented in the main text.

Considering the previous examples of image processing, including this example, we can conclude that disk-shaped SE is an extremely versatile and useful SE.

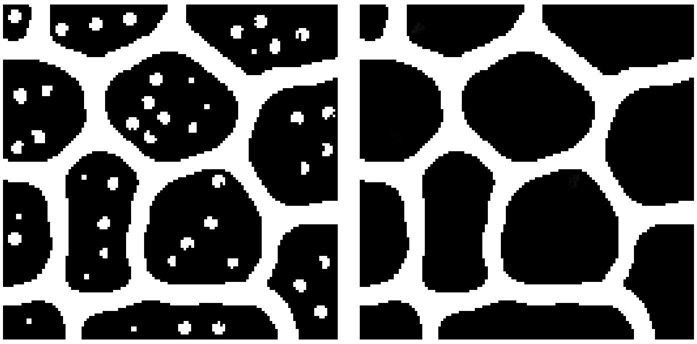

Fig. R13: For simple images (binary image), RMP opening using line-shaped SE is effective. However, in practice, processing images with complex intensity distributions is necessary.

Comment 13: *L187: same as L86; can be simplified in "... and the number of image rotations on ..."*
Author response: The relevant sentence has been changed (p. 12, lines 204–205).
