# Supplementary material for: RMPJ: An ImageJ plugin for morphological information processing in biomedical images
Source: PLoS Comput Biol. 2025 Apr 16;21(4):e1012992. doi: 10.1371/journal.pcbi.1012992 (PMC12037072; doi:10.1371/journal.pcbi.1012992)
Supplement: Attachment — Submitted filename: Response_Letter.docx [file pcbi.1012992.s004.docx]

**Response to the reviewers**

I would like to thank the reviewers for their helpful comments and suggestions for improving my paper “RMPJ: An ImageJ plugin for morphological information processing in biomedical images” (Ms. No.: PCOMPBIOL-D-24-00594). I have revised the manuscript based on the feedback from both reviewers. Below are the point-by-point responses to their comments, along with the changes made in the manuscript. Reviewers’ comments are quoted text in italics.

The figure that was “Fig. 1” in the first manuscript has been deleted. Please note that the figure numbers have been changed in the revised manuscript from the first manuscript.

**The comments of the reviewer #1 and author response**

Thank you for evaluating this paper. I appreciate you taking the time to extensively review it. Below are my responses to your questions and comments.

**Comment 1:** *How was the original image in Fig. 2a produced? (Original samples used in other figures are clearly explained).*

**Author response: (**Because Fig. 1 in the first manuscript was deleted, the figure that was “Fig. 2” in the first manuscript was changed to “Fig. 1” in the revised manuscript.) The original image in Fig. 1(a) is a synthetic image created by the author to confirm the effect of the opening operation due to changes in the shape of the SE and the number of rotations of the image.

This explanation has been added to the revised manuscript (p. 11, lines 192-194).

**Comment 2:** *Some things and parameters need to be determined to perform RMP-based processing, shape of the structuring element (SE), its size, number of iterations, etc. Does the author have any guidance on how to achieve a correct result other than trial and error?*

**Author response:** The process for determining the size of SE is similar to the traditional mathematical morphology. The size is determined by considering the structural characteristics of the object being analyzed. When applying operations such as opening to smooth an image, an SE slightly larger than the structures to be removed, such as noise, should be used. For image enhancement filters (such as top-hat transforms), the SE should be slightly larger than the domain of the structure to be enhanced. This is because structures smaller than the size of the SE are removed first, and then those structures are enhanced.

In RMP, when determining the shape of the SE, the line shape is used to process thin line structures in images and extract granular shapes. The square or disk shape is more commonly used. In many cases, the processing results are accurate when a disk shape is used rather than a square shape.

Empirically, for the number of iterations *N*, the optimal value for disk-shaped SE and square-shaped SE is *N*=8, and for line-shaped SE, *N*=36 is optimal.

An additional experiment was performed to quantitatively show the effect of morphological processing owing to differences in the shape of SE and the value of *N*. The details of this experiment are provided in Section 2.4. of the S1 Text.

**Comment 3:** *The author demonstrates a good application result for stacked image data. For such serial image data, does the author think there is a need to extend the 2D SE to a 3D SE (voxel type) in the future?*

**Author response:** Yes. It is necessary to extend the current theory to enable the use of RMP with voxel data SE (3DSE). Rather than processing objects with a 3D structure as 2D slice images, allowing the voxel data of the analysis target to be processed would be advantageous for noise removal and morphological feature extraction. This would enhance the process wile considering the connected information of the 3D structure.

However, in this case, the voxel data to be processed is rotated relative to the 3DSE. A significant issue arises as the computational cost increases substantially when performing morphological analysis while rotating the object in 3D. Future work will focus on developing methods to process the data at a practical speed.

**The comments of the reviewer #2 and author response**

I appreciate the detailed and helpful comments provided. I agree with the reviewer’s observation that the introduction lacks a thorough explanation of RMP. Following this suggestion, I have revised the Introduction section to first highlight the limitations of traditional mathematical morphology, followed by an introduction to the general concept of RMP, and concluding with an explanation of its advantages (pp. 4-6, lines 70-96).

I have responded to the other comments as follows.

**Comment 1:** *How does RMP relate to path opening methods developed by Heijmans et al.? It seems to me there may be similar objectives.*
**Author response:** The path opening method was devised to extract linear features of arbitrary curves from images; however, in some cases, the same objective can be achieved using RMP-based operations.

Three main methods exist for extracting elongated (thin) objects from images using mathematical morphology-based processing: (1) using line-shaped SE, (2) using area and other morphological attributes, and (3) path opening.

Path opening was proposed to solve the problems of methods (1) and (2). For example, with traditional mathematical morphology-based operations, it can be challenging to obtain satisfactory results even with linear-shaped SE when the processing target object consists of curves or line segments with multiple directions.

RMP-based operations are included in method (1). However, While RMP also uses linear SEs, it rotates images, allowing it to handle thin objects with arbitrary shapes more effectively than traditional mathematical morphology-based processing.

To demonstrate the applicability of RMP to thin structure extraction, an example of extracting the crack region on the road surface (represented as a thin filament-like structure in the image) has been added to the revised S1 Text (Section 2.3. of S1 Text).

Comparing the performance of RMP with that of path opening in extracting thin structures would be a valuable future topic for investigation. I would like to work on this as a future issue.

**Comment 2:** *From a technical point of view, would it make a big difference to apply the rotation to the SE instead of to the image? I suppose this is a question of discretisation issue?*

**Author response:** Yes. The reason for rotating the image is to avoid problems arising from the discretisation of the SE. When a rotation transformation is applied to a small shape such as an SE at an arbitrary angle, the SE does not retain its original structure after the rotation.

The image below Fig. R1(a) is a 5×5 pixels square (assuming a square-shaped SE). Fig. R1(b) is an image of Fig. R1(a) rotated 22.5° clockwise. A bi-linear interpolation method was used for the rotation transformation. The resultant image of applying binarization to this rotated image is Fig. R1(c). From this result image, the square shape has not been maintained owing to the rotation transformation. Fig. R1(d) is the result of rotating the image in R1(a) 22.5° without any interpolation and then converting the rotated image to binary.

From this example, there is a concern that using SE that has undergone a rotation transformation in morphological processing will negatively influence the processing results. Thus, instead of rotating the SE, the RMP uses a strategy that rotates the image being processed.

Because the image to be processed is also discrete, the structures within it are also damaged by the rotation transformation. However, by applying an appropriate interpolation method and combining multiple rotated images, this discretisation problem can be reduced.

Fig. R1(e) shows a yellow 5×5 pixels square object (left) and an object (right) created by rotating the square object 45°, both placed on a background with noises. The result of applying the RMP-based opening to this image using a 3×3 pixels square-shaped SE is Fig. R1(f) (*N* = 8). Considering the results of the opening based on RMP, the shapes of the structures in the foreground are retained, and only the noise is removed. In addition, Fig. R1(g) shows the result of the traditional opening. The structure of the right object is deformed when the traditional opening is applied.

**Fig. R1.** (a) An image containing a 5x5 pixels square object. As it is an 8-bit binary image, the intensity value for black is 0 and the intensity value for white is 255. (b) An image created by rotating (a) clockwise by 22.5°. As a rotation transformation with interpolation (bi-linear method) was used, pixels with intensity values other than 0 and 255 were created at the boundary of the object. (c) The binary image of (b). (d) The image of (a) rotated 22.5° without interpolation. (e) The two objects shown in yellow are the objects to be processed. The size of the object on the left is 5 × 5 pixels. The object on the right was created by rotating the object on the left by 45°. In addition, the structures expressed in white that are placed in the background are considered to be noise. (f) The result of applying an RMP-based opening using a 3 × 3 pixels square SE. (g) The result of a traditional opening.

**Comment 3:** *Would it be possible to extend the method to the processing of 3D images by applying 3D rotation?*

**Author response:** I think it is possible to extend the RMP method to 3D images (voxel data). However, as aforementioned (in response to comment 3 in reviewer #1), the problem is that the computational cost for the 3D rotation and subsequent 3D morphological processing of the analysis target is high. As a future issue, I would like to develop a processing method that reduces the computational cost.

**Comment 4:** *Finally, the proposed RMP framework looks promising. Beyond the presentation of the RMPJ software, I wonder is a methodological article could have been more appropriate?*

**Author response:** All morphological operations based on the seven types of RMP implemented in RMPJ are derived from previously published methods (including ${MCE}_{1}$ and ${MCE}_{2}$, which are described below). Therefore, I have decided to write a paper on the software that implemented them, as I believe that I could not demonstrate novelty in terms of methodology.

In addition, I have received requests from my co-researchers and readers of my published papers to create software for RMP. To meet this demand and further promote RMP, it is necessary to publish a detailed explanation of RMPJ as a software paper.

**Comment 5:** *L45: maybe pixel by pixel is more appropriate?*
**Author response:** I agree with the reviewer’s suggestions. I have revised the main text based on the suggestions (p. 3, line 50).

**Comment 6:** *L47: a quick explanation of what are opening, closing and top-hats would make the reading more comfortable.*
**Author response:** A brief explanation of opening, closing, and top-hats has been added (pp. 3-4, lines 53-62).

**Comment 7:** *L72: the software propose rotational version of classical operators, as well as new operators (MCE1 and MCE2).
This can be better emphasized.*

**Author response:** ${MCE}_{1}$ and ${MCE}_{1}^{R}$ are proposed by Kimori (2011) (main text reference no. [7]). The ${MCE}_{2}$ algorithm is formalized in Soille’s textbook (main text reference no. [3]), and ${MCE}_{2}^{R}$ is proposed by Kimori (2013) (main text reference no. [8]).

Citations were made for these operations in the main text (p. 10, lines 163-167) and S1 Text (second paragraph from the bottom of p. 3 and last paragraph on p. 7). In addition, some explanations of the characteristics of both operations (${MCE}_{1}^{R}$ and ${MCE}_{2}^{R}$) have been added to the main text (p. 10, lines 163-167).

**Comment 8:** *L90: It would be nice to give a short explanation of what do the different operators* do.
Typically in the introduction, or in the beginning of the Design section.

**Author response:** In the “Design and Implementation” section (p.8, lines 128-136), brief explanations of the seven operators have been added.

**Comment 9:** *L103: I am surprised that an optimum value for line SE exist. I would have expected it to depend on the application, typically on the curvature or thickness of the structures to filter.*

**Author response:** The optimal value of *N* (number of iterations) when using a line-shaped SE is 36 (see S1 Text, section 2.4.). In this case, the image is rotated in 5° increments. Even if there are structures with various orientations in the image, it is believed that most structures with various angles can be processed if the processing is performed for each increment of this angle as well as if the image is rotated and processed in small increments of 5°.

The optimum value of this SE length depends on the structure being processed, as it must be selected based on the structure’s characteristics (curvature and thickness). This is consistent with traditional mathematical morphology.

**Comment 10:** *L124-127: part of this paragraph could be presented in the introduction.*

**Author response:** The sentences have been moved to the “Introduction” section (p. 6, lines 92-96).

**Comment 11:** *Fig.2: I am surprised that the result of WTH and WTH^R with disk differ. As the disk is (in continuuous domain at least) rotation invariant, I would expect to obtain the same result.*

**Author response:** (In the revised manuscript, “Fig. 2” in the first manuscript was changed to “Fig. 1”.) The shape of the disk, expressed discretely, does not necessarily have rotational symmetry at any given angle. It only has rotational symmetry at 90°, 180°, 270°, and 360°.

Fig. R2 shows a specific example of the effect of the shape of discrete SE on morphological processing. The upper part of Fig. R2(a) is the original image to be processed, and the lower part is a disk-shaped SE with a diameter of 11 pixels. The opening process was performed on the original image using this SE. The result of the traditional opening operation is shown in the upper part of Fig. R2(b). The lower part is an image that expresses the result of the upper part in pseudo color. This demonstrates that the circular objects in the original image have been deformed by the shape of the SE. This is because isotropic processing cannot be performed on the structure of the analyzed object when using a discretely represented disk shape.

This characteristic of traditional morphological operations was identified as a weakness, which motivated the development of RMP. In various research fields, image processing is not typically the final stage of the research process. In many cases, analysis (measurement) using the processed image is performed following the image processing process. Therefore, if artifacts based on the shape of the SE occur, serious problems affecting the analysis results will occur.

Fig. R2(c) shows the result of the opening operation based on RMP. Even when using discrete SE, the processing that preserves the structure of the analysis target in the original image has been achieved. Furthermore, the outline shape of the circular object and the dark regions of low intensity within the object region are preserved.

**Fig. R2**. (a) The upper panel shows the original image. The lower panel shows the disk-shaped SE with a diameter of 11 pixels. (b) The upper panel shows the result of the traditional opening. The lower panel shows the pseudo-color display of the opening image in the upper panel. (c) The result of the opening based on RMP.

**Comment 12:** *L169+: again, I do not understand the interest of using RMP with a disk structuring element?*

**Author response:** To extract the filament structure, it is necessary to determine the width of the filament domain to be extracted. This width must be regulated by the diameter of the disk-shaped SE.

If the intensity is considered to be height, the image can be considered a 3D topographical map. For example, the grayscale image shown in Fig. R3(a) is displayed in 3D, as shown in Fig. R3(b). The filament structure can be considered as having a shape like a mountain range. Emphasizing the filament structure corresponds to extracting and emphasizing the upper part of the mountain range. To emphasize this, white top-hat transform is used. In this case, the domain at the upper part of the mountain range with a width narrower than a certain size is extracted. To process this, a disk-shaped SE is required. The width of the topmost domain of the mountain range to be extracted is specified by the diameter of the disk-shaped SE.

Fig. R3(c) shows the intensity distribution along the dotted line in Fig. R3(a), which is the original image $f$. The results of the opening $\left( \gamma^{R}\left( f \right) \right)$ with the disk-shaped SE are shown by the black dotted line in Fig. R3(c). The length of the diameter of the disk-shaped SE is denoted by $d$. The ${WTH}^{R}$(i.e. the operation of subtracting the opening image $\gamma^{R}\left( f \right)$ from the original image $f$ ) extracts the residual structures (shown in purple in Fig. R3(c)). Fig. R3(d) is the result of applying the ${WTH}^{R}$ using a disk-shaped SE to Fig. R3(a).

However, in the RMP-based opening process, the image is rotated; thus, the line-shaped SE fits most of the mountain range structure. Therefore, the ${WTH}^{R}$ using that SE cannot extract the structure of the upper part of the mountain range. Fig. R3(e) shows the result of applying the ${WTH}^{R}$ using line-shaped SE to Fig. R3(a). During the opening process based on RMP, the line-shaped SE fit the structure of the mountain range; thus, the shape of the original image was preserved (that is, ${f=\gamma}^{R}\left( f \right)$). Therefore, no residual region is generated in the ${WTH}^{R}$, and consequently, no structure is extracted.

However, if this line-shaped SE (if it is used in operations based on RMP) makes use of its feature of ‘fitting into mountain ranges’, it may be possible to extract the structure of mountain ranges using smoothing operations. By employing the opening operations, it becomes possible to remove the surrounding structure (noise, etc.) that does not fit the SE while preserving the structure of the mountain range that does fit the SE. If the smoothed image is converted to a binary image, it is expected that the mountain range structure will be separated from the background.

The results of applying this method to the extraction of crack regions in road pavement are shown in Section 2.3 of S1 Text. However, this method is only effective when a high contrast exists between the filament and background regions. Otherwise, it is thought that the filament region cannot be separated from the background owing to binarization.

**Fig. R3.** (a) Grayscale image, which includes filament structures. (b) 3D representation of (a). (c) Intensity distribution along the dotted line in (a). (d) Result of applying the ${WTH}^{R}$ using the disk-shaped SE to (a). (e) Result of applying the ${WTH}^{R}$ using the line-shaped SE to (a).

**Comment 13:** *L246: GitHub link is not active...*

**Author response:** The source code has been re-saved in the public repository on GitHub.

**Comment 14:** *S1: the MCE1 and MCE2 operators seems to be new. I suggest to present them more in details in the main part of manuscript.*

**Author response:** As aforementioned (in response to comment 7), I have cited the literature in which these operations. The cited literature provides detailed explanations of the theory and application cases of these operations.

**Comment 15:** *S1, section 1.2: I strongly disagree with the sentence "the constant scanning direction of the SE
during the morphology operation causes the deformation of the shape of the object."
The scanning order has nothing to do with the deformation of the objects. The deformation is caused solely by the shape of the SE. The same result is obtained when scanning the image backwards, or in random order. Please correct.*

**Author response:** I have corrected the relevant parts (the first paragraph of section 1.2. of S1 Text) based on the reviewer’s instructions. I have also added a more detailed explanation of the deformation of the target shape caused by the shape of the SE and revised Fig. S2 in S1 Text.

**Comment 16:** *S1. I am surprised not to see an example based on the enhancement of filament-like structure, similar to what was presented in Kimori et al, 2016. To my opinion, this is one of the main use-cases of the proposed method!*

**Author response:** There is a significant demand to extract filament structures. As you mentioned, I agree that this is the main use case for RMP.

In this study, I have included examples that emphasize filament structures such as mammary glands (Fig. 2 in the main text) and filamentous structures within cells (Fig. S6 in the S1 Text). Additionally, in the revised manuscript, it was emphasized that the primary purpose of applying RMP is to emphasize filament-like structures in the “Results” section (pp. 13-14, lines 234-237).

In this section, I have also added a note that cited Kimori et al. (2016) (main text reference no. [6]). It is possible to perform structural analysis of filament-like structures and directional analysis of filaments by using RMP.

In addition, as an example of related image processing, the extraction of road crack structures using line-shaped SE has also been added in Section 2.3. of S1 Text.
